# Suppression of MAPK11 or HIPK3 reduces mutant Huntingtin levels in Huntington's disease models

**DOI:** 10.1038/cr.2017.113

**Published:** 2017-10-13

**Authors:** Meng Yu, Yuhua Fu, Yijiang Liang, Haikun Song, Yao Yao, Peng Wu, Yuwei Yao, Yuyin Pan, Xue Wen, Lixiang Ma, Saiyin Hexige, Yu Ding, Shouqing Luo, Boxun Lu

**Affiliations:** 1 State Key Laboratory of Medical Neurobiology, Huashan Hospital, School of Life Sciences, Collaborative Innovation Center for Genetics and Development, Fudan University, Shanghai 200438, China.; 2 Department of Anatomy and Histology & Embryology, Shanghai Medical College, Fudan University, Shanghai 200032, China.; 3 Peninsula Schools of Medicine and Dentistry, Institute of Translational and Stratified Medicine, University of Plymouth, Research Way, Plymouth, PL68BU, UK.

**Keywords:** PolyQ, high-throughput screening, protein homeostasis, kinase, positive feedback mechanism, neurodegenerative disorders

## Abstract

Most neurodegenerative disorders are associated with accumulation of disease-relevant proteins. Among them, Huntington disease (HD) is of particular interest because of its monogenetic nature. HD is mainly caused by cytotoxicity of the defective protein encoded by the mutant *Huntingtin* gene (*HTT*). Thus, lowering mutant HTT protein (mHTT) levels would be a promising treatment strategy for HD. Here we report two kinases HIPK3 and MAPK11 as positive modulators of mHTT levels both in cells and *in vivo*. Both kinases regulate mHTT via their kinase activities, suggesting that inhibiting these kinases may have therapeutic values. Interestingly, their effects on HTT levels are mHTT-dependent, providing a feedback mechanism in which mHTT enhances its own level thus contributing to mHTT accumulation and disease progression. Importantly, knockout of *MAPK11* significantly rescues disease-relevant behavioral phenotypes in a knockin HD mouse model. Collectively, our data reveal new therapeutic entry points for HD and target-discovery approaches for similar diseases.

## Introduction

Neurodegenerative disorders are devastating diseases caused by progressive loss of neurons in the central nervous systems. They influence millions of people, especially in the aged population. Treatment of such diseases has been extremely challenging, due to the complexity of their diagnosis and pathogenic mechanisms^1^. On the other hand, many types of neurodegenerative disorders share the common feature of the accumulation of misfolded and aggregation-prone proteins, and lowering the levels of these proteins is considered as an appealing therapeutic strategy^2^. This strategy is likely to be more effective in inherited monogenetic neurodegenerative diseases, because the casual relationships between the mutant proteins and the diseases are clear: the mutant proteins encoded by these mutated genes are more likely to be the causes rather than the consequences of the diseases. In addition, the patients can be diagnosed earlier, offering the possibility of preventative treatment.

Thus, Huntington's disease (HD) represents an important model for neurodegenerative disorders because of its monogenetic nature. HD is caused by the mutation of the *Huntingtin* (*HTT*) gene, which encodes the mutant HTT protein (mHTT) with an expanded polyglutamine tract (polyQ)^3^. The gain of toxic function of mHTT is the major cause of HD, but the molecular mechanisms are unclear and likely involve multiple pathways^4^, which are highly challenging to target simultaneously. On the other hand, lowering mHTT protein levels has been shown to effectively mitigate mHTT toxicity: in an HD mouse model expressing inducible mHTT N-terminal fragments, turning off transgene expression reversed neuropathology and motor deficits^5^; in several mammalian models, delivery of short hairpin RNAs or small interference RNAs (siRNAs) or antisense oligonucleotides (ASOs) against mHTT attenuate neuropathology and disease-related phenotypes^6,7,8,9^. Screening studies also revealed genetic and chemical modifiers that reduce mHTT aggregates and rescue HD-relevant phenotypes^10^. Recent studies have also shown that the level of soluble mHTT is closely related with disease pathology^11,12,13^, and thus lowering soluble mHTT may provide an effective approach to treat HD by ameliorating any downstream toxicity. If successful, this strategy is able to modify disease progression, and similar strategies could be applied to other diseases as well^14^. Previous mHTT-lowering studies are mostly based on RNA interference (RNAi) or ASOs. However, it is challenging to deliver such reagents to the affected brain regions of HD patients. Thus, druggable targets for low-molecular weight compounds that can lower mHTT levels are highly desired. Meanwhile, the wild-type HTT protein (wtHTT) is an essential protein during development^15,16^ and may also play a role in HD^17^ and lowering wtHTT to an intolerable extent may have a harmful impact. Thus, the wtHTT levels and phenotypic outcomes need to be carefully evaluated in any strategy based upon lowering of HTT levels.

Here, we report an unbiased RNAi screen that reveals the kinases MAPK11 and HIPK3 as novel modulators of mHTT protein levels. Both kinases influence mHTT levels via their kinase activities; opening up new drug discovery opportunities. We further validated the effect of MAPK11 and HIPK3 *in vivo* in an HD knockin (KI) mouse model, and confirmed their therapeutic potentials in lowering mHTT levels.

## Results

### Unbiased screen reveals genetic modulators of mHTT levels

**Figure 1:**
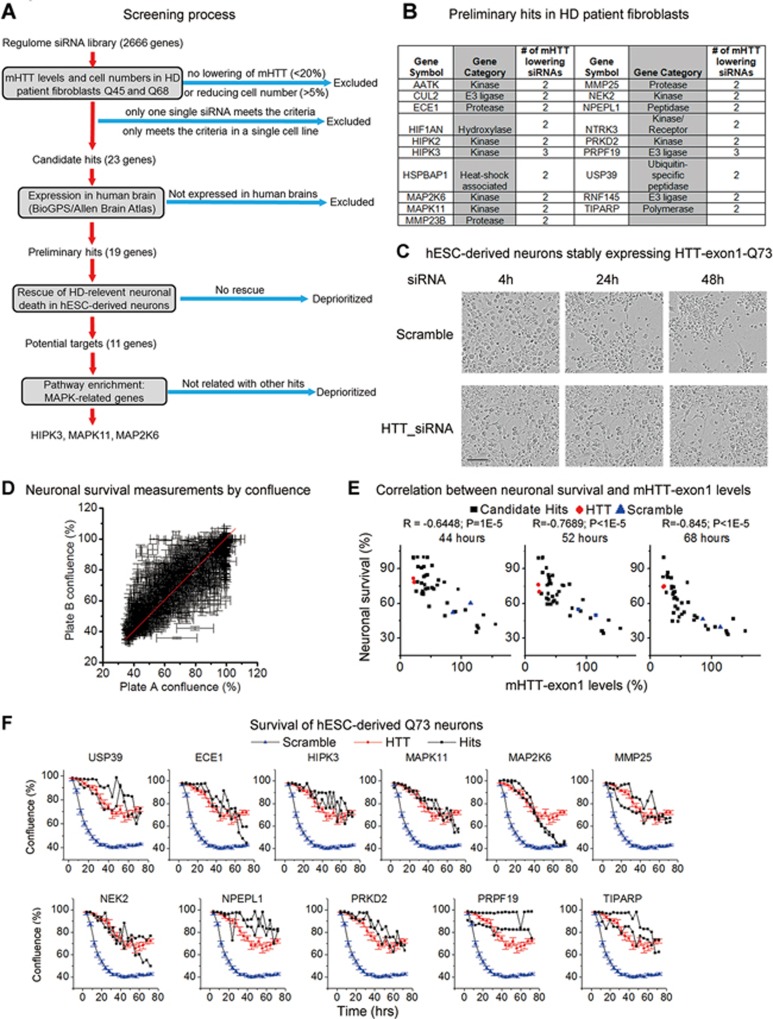
Potential druggable genetic modifiers of mHTT levels identified by screening. **(A)** A schematic flowchart showing the screening process. **(B)** The information of potential preliminary hits. mHTT levels were measured by HTRF using the 2B7/MW1 antibody pair in two different human HD patient fibroblast lines (Q68 versus Q45). “# of mHTT lowering siRNAs” indicates the number of siRNAs (out of four) that reduce mHTT levels in both lines. **(C)** When cultured under standard (non-protective) conditions, hESC-derived neurons stably expressing HTT-exon1 fragments exhibited a long polyQ (Q73) specific degeneration phenotype, which could be assessed by imaging-based measurements. Representative images (of over 20 biological replicates) show the neuronal survival changes in these cells (Q73 neurons) transfected with scrambled siRNA (scramble) or HTT-exon1 siRNA (HTT). Scale bar, 50 μm. **(D)** The candidate hits were tested in Q73 neurons using SMART-pool siRNAs (Dharmacon) and the confluences measured by imaging at different time points were calculated by IncuCyte based on four fields in each well. The signals from two independent transfections (Plate A versus B) show consistency, signals are clustered near the diagonal line (red). **(E)** In Q73 neurons transfected with siRNA-pools targeting the primary hits, the averaged confluences of wells transfected with each siRNA in each plate were plotted with the mHTT-exon1 levels (eight measurements from two independent transfected wells) and measured by HTRF using the 2B7/MW1 antibody pair 48 h after transfection. The correlation coefficient *R* and *P* values for confluences measured at 68 h were calculated by Spearman correlation analysis. **(F)** Neuronal survival plots of Q73 neurons transfected with indicated siRNAs. Neuronal survival was measured by the averaged confluence of four fields in each well. Note that the scramble and the HTT siRNA signals of all these plots are from the same samples tested in parallel with the candidate genes. The scrambled (*n* = 16) and HTT siRNA (*n* = 4) plots represent mean and SEM, whereas each of the two independently transfected wells of the hits are plotted individually. The genes targeted by siRNAs that obviously increased neuronal survival (higher survival at all the time points measured) were selected as potential hits.

To reveal druggable modulators of mHTT levels, we performed an RNAi screen (Figure 1A) using a focused siRNA library (regulome) targeting 2 666 genes expressing proteins (mostly enzymes and receptors) that belong to the protein families that are capable of regulating protein levels. The screen was performed in immortalized HD patient fibroblasts from two independent patients (Q45 and Q68) expressing endogenous full-length human mHTT proteins. mHTT levels were measured by the homologous time-resolved fluorescence (HTRF) assay using the 2B7/MW1 antibody pair which selectively detects mHTT^18^ (Supplementary information, Figure S1). The HTRF assay utilizes a terbium-conjugated antibody (donor) and a D2-conjugated antibody (acceptor) targeting the same protein; time-resolved fluorescence resonance energy transfer^19^ occurs when the two antibodies come into close proximity by binding with a common protein molecule. As a result, the HTRF signals are proportional to the target protein concentration and can be used to quantify its level^20^. This technology has been successfully applied to the measurement of HTT levels in previous studies^21,22^.

The genes with at least two (out of four) independent siRNAs that reduced mHTT more than 20% in both lines without reducing the cell number by more than 5% (measured by cell titer-glo) were selected as candidate hits. Genes that are not expressed in the human brain based on the BioGPS database^23^ and the Allen Brain Atlas^24^ were excluded. The remaining preliminary hits (Figure 1B) were then tested in a human neuronal HD model^13^ for phenotypic validation (Figure 1C-1F). These neurons were derived from human embryonic stem-cells (hESC) stably expressing mHTT-exon1 (Q73) fragment and exhibited a mHTT-dependent neuronal loss phenotype (measured by cell confluence) under standard non-protective culture conditions^13^ (Figure 1C). The preliminary hits were tested by transfecting siRNA-pools targeting these hits in 96-well plates with biological duplicates (Figure 1D). The cultures exhibited substantial differences in neuronal survival, which negatively correlated with the mHTT-exon1 Q73 levels measured by HTRF (Figure 1E). This is consistent with the expectation that changing mHTT levels via the modifiers influences the survival of HD neurons. 11 out of the 19 preliminary hits showed obvious rescue of survival in this neuronal model (Figure 1F).

### MAPK11 and HIPK3 regulate endogenous mHTT levels

Among the 11 identified putative mHTT modulators, we found a clustering of MAPK-related proteins: MAP2K6 and MAPK11 that belong to the same signaling pathway, in which MAP2K6 phosphorylates MAPK11^25^; HIPK3, which is regulated by JNK1 and JNK2 (also referred to as MAPK8 and MAPK9)^26^. The enrichment of genes for MAPK-related proteins suggested that these kinases may have a higher chance of being reliable mHTT modulators and we therefore prioritized MAPK11, MAP2K6 and HIPK3 for further validation.

**Figure 2:**
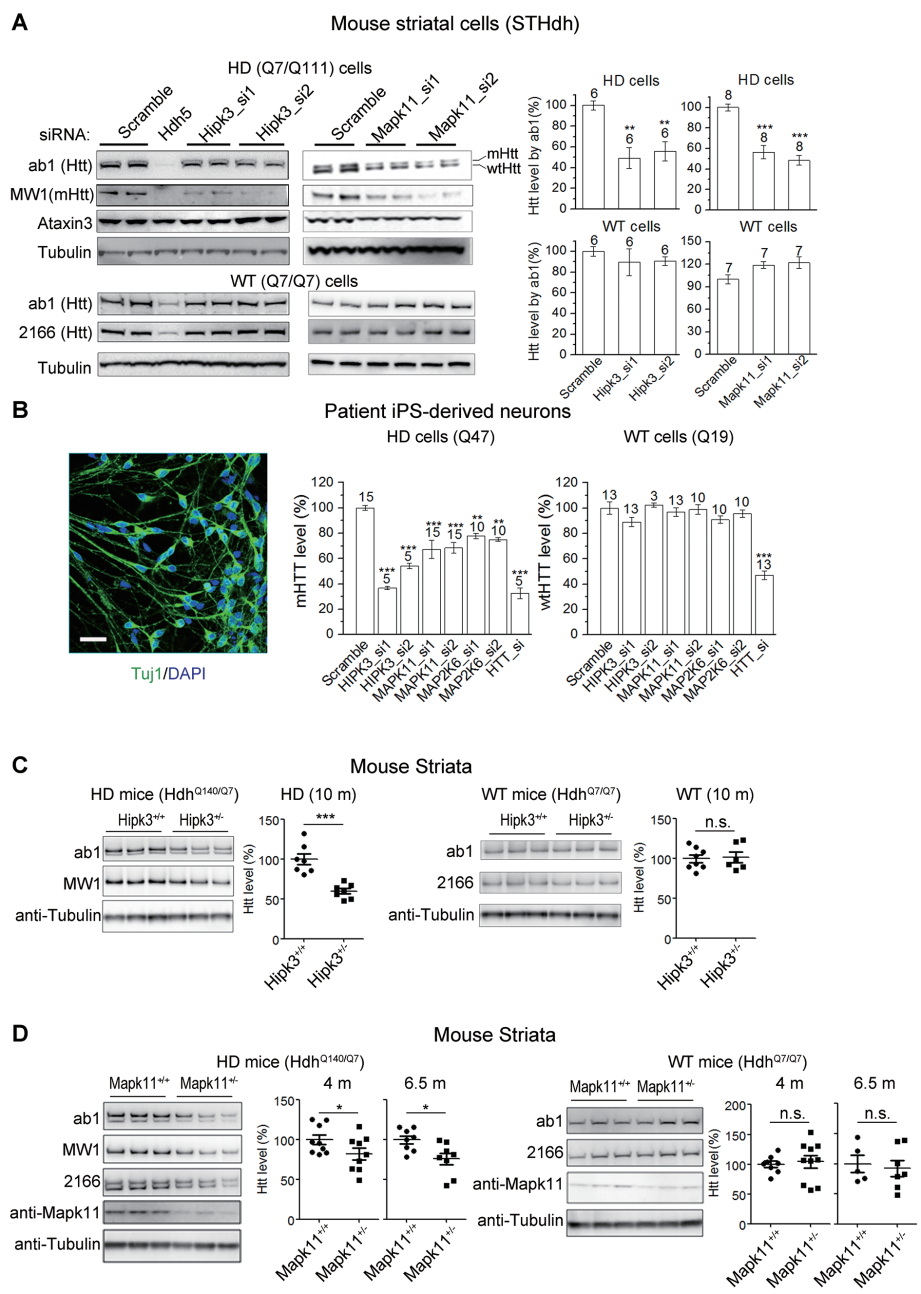
HIPK3 and MAPK11 regulate HTT levels. **(A)** Left panels, representative western blots for the indicated proteins in *STHdh* cells upon transfection of the indicated siRNAs. Note that ab1 (N terminal antibody) detects both mHtt and wtHtt, and MW1 (polyQ antibody) detects mHtt only. Knocking down *Hipk3* or *Mapk11* reduces Htt levels in the HD (Q7/Q111) cells but not the WT (Q7/Q7) cells; Right panels, quantifications of the western blots. *Hdh5* is an siRNA directly targeting the mouse *Htt* gene. **(B)** Left, A representative image showing the HD patient iPSC-derived neurons (blue, DAPI; green, Tuj1; scale bar, 50 μm); middle, mHTT levels of the HD patient iPSC-derived neurons measured by HTRF using the 2B7/MW1 antibody pair that detects mHTT specifically. Right, wtHTT levels of the control patient iPSC-derived neurons measured by HTRF using the 2B7/2166 antibody pair. **(C)** Representative western blots and quantifications showing that heterozygous deletion of *Hipk3* reduces endogenous Htt levels in the striata of 10-month-old HD (*Hdh*^Q140/Q7^) mice, but not WT (*Hdh*^Q7/Q7^) mice. For quantifications, the Htt signal (measured by ab1) were first normalized to the loading control (β-Tubulin). The loading control corrected signals were then normalized to the average of WT controls at the same age. **(D)** Representative western blots and quantifications showing that heterozygous deletion of *Mapk11* reduces endogenous Htt levels in the striata of 4- and 6.5-month-old HD (*Hdh*^Q140/Q7^) mice, but not in WT (*Hdh*^Q7/Q7^) mice. The quantification is the same as **(C)**. For all bar plots in this figure, all signals were normalized to the average signal of the scrambled siRNA-transfected controls; the bars were plotted as mean and SEM; the number on top of each indicates the number of biological replicates, and the statistical analysis was performed by one-way ANOVA and *post hoc* Dunnett's tests for **A** and **B**, and two-way unpaired *t* tests for **C** and **D**, compared to the scramble siRNA-transfected control. ^*^P < 0.05; ^**^P < 0.01; ^***^P < 0.001; ns, *P* > 0.05.

We first validated these targets in the mouse HD striatal cell line *STHdh*^Q7/Q111^^27^, a well-established cellular model which expresses endogenous full-length mHtt (the mouse homolog of human mHTT) with 111Q. The HTRF assay utilizing the 2B7/MW1 antibody pair does not work properly in *STHdh* cells. Moreover, the 2B7/2166 antibody pair works well but detects the total signals from both mHtt and the wtHtt^28^. Thus, we first validated these targets by western blots instead of using HTRF in *STHdh* cells. Knocking down these targets using siRNAs (Supplementary information, Figure S2A-S2C) significantly reduced the endogenous level of soluble full-length mHtt (Figures 2A and 4A) in HD cells (Q7/Q111). The reduction in mHtt did not appear to result from cleavage changes, because no increase of mHtt fragments at lower molecular weights was observed (Supplementary information, Figure S2D).

To further validate these targets in HD models in which HTT has a polyQ length equivalent to that more commonly found in HD patients, we tested these targets in Q47 human induced-pluripotent-stem cell (iPSC)-derived neurons expressing endogenous mHTT with 47Q^29^. We chose to use the 2B7/MW1 HTRF assay (Supplementary information, Figure S1), which is a more accurate and reliable way of measuring mHTT in these cells^29,30^. Knocking down these targets by siRNAs (Supplementary information, Figure S2A-S2C) reduced the endogenous soluble mHTT levels in these iPSC-derived neurons (Q47 neurons) (Figure 2B), confirming their value in the human HD neuronal model expressing mHTT with 40-50Q, a polyQ length most commonly found in HD patients.

We then triaged the targets by validating their effects on mHtt levels in mouse models *in vivo*. MAP2K6 is an upstream activator of MAPK11, its major downstream effector. Thus, MAP2K6 may regulate mHTT through MAPK11. In accord with this, knockdown of *Mapk11* diminished the Map2k6-mediated effect on mHTT levels in *STHdh*^Q7/Q111^ cells (Supplementary information, Figure S3A). This epistasis effect is in agreement with Mapk11 being downstream of Map2k6. In addition, *Map2k6* knockout results in immune defects and delay in testis determination^31^. Therefore, considering that *in vivo* validations are highly time-consuming and resource-consuming, we prioritized MAPK11 and HIPK3 for *in vivo* validation.

**Figure 3:**
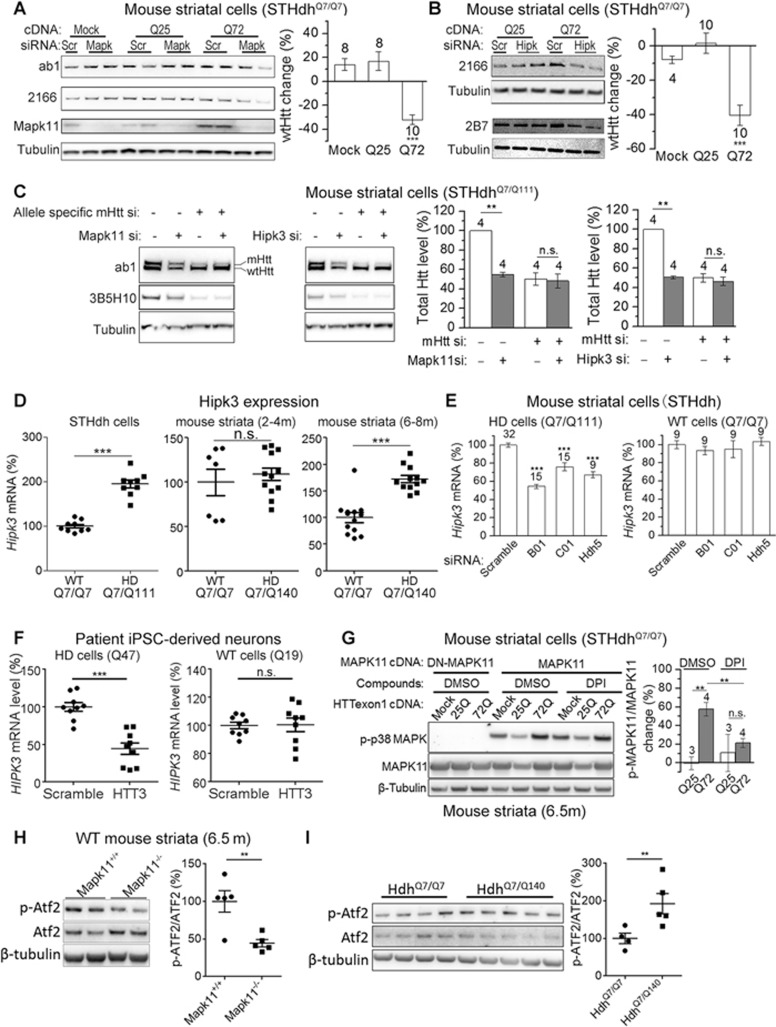
HIPK3- and MAPK11- mediated HTT regulation is mHTT-dependent. **(A)** Representative western blots and quantification of the *STHdh*^Q7/Q7^ (WT) cells showing that expression of the mHTT (Q72) but not the wtHTT (Q25) N-terminal exon1 fragments enables the reduction of endogenous wtHtt levels by knocking down *Mapk11*. The wtHtt change by the siRNA targeting *Mapk11* was calculated within each group of cDNA transfected samples and plotted as the percentage difference between each of the *Mapk11* siRNA-transfected well and the average of the scramble siRNA-transfected control within the same group. The statistical analysis was performed by one-way ANOVA and Dunnett's *post hoc* tests. **(B)** Similar to **(A)**, but showing the effects of knocking down *Hipk3*. **(C)** Representative western blots and quantifications (by ab1) of the HD (*STHdh*^Q7/Q111^) cells showing that specifically knocking down *mHtt* using the allele-specific siRNA (allele specific mHtt si) abolished the reduction of endogenous Htt levels by knocking down *Mapk11* or *Hipk3*. The statistical analysis was performed by two-tailed unpaired *t* tests. **(D)** qPCR measurements of *Hipk3* mRNA levels in the HD (Q7/Q111) versus the WT (Q7/Q7) *STHdh* cells (left), and in the HD (*Hdh*^Q140/Q7^) versus the WT (*Hdh*^Q7/Q7^) mouse striata of animals at indicated ages (middle and right). The levels were normalized to the average of WT samples. The statistical analysis was performed by two-tailed unpaired *t* tests. **(E)** qPCR measurements of *Hipk3* mRNA levels in the HD (*STHdh*^Q7/Q111^) cells or the WT (*STHdh*^Q7/Q7^) cells transfected with the indicated siRNAs. B01, C01 and Hdh5 are siRNAs targeting *Htt* (see Supplementary information, Data S1). The levels were normalized to the average of scrambled siRNA-transfected control samples (Scramble), and the statistical analysis was performed by one-way ANOVA followed by *post hoc* Dunnett's tests. **(F)** Similar to **(E)**, but in patient iPSC-derived neurons. HTT3 is the siRNA targeting human *HTT*. **(G)** Representative western blots and quantifications of phospho-MAPK11/MAPK11 ratio in *STHdh*^Q7/Q7^ WT cells transfected with indicated cDNA and treated with indicated compounds. The p-MAPK11/MAPK11 ratio change compared to the Mock control was quantified to assay the activation of MAPK11. **(H)** Western blots and quantification of Atf2 and phosphorylated Atf2 (p-Atf2) levels in striata lysates from 6.5-month-old mice with or without *Mapk11* knockout in the wild-type background using specific antibodies targeting Atf2 versus phosphorylated Atf2, respectively. Atf2 phosphorylation was significantly decreased in *Mapk11* knockout mice, confirming Atf2 as a substrate for Mapk11. Statistical analysis was performed by two-tailed unpaired *t* tests. **(I)** Similar to **(H)**, but comparing HD (*Hdh*^Q7/Q140^) versus WT mice (*Hdh*^Q7/Q7^). Atf2 phosphorylation was significantly increased in HD mice, suggesting that Mapk11 activity may be increased. For all plots, error bars represent mean and SEM. ^*^P < 0.05; ^**^P < 0.01; ^***^P < 0.001; ns, *P* > 0.05.

We confirmed that MAPK11 and HIPK3 are expressed in both human and mouse brains (Supplementary information, Figure S3B-S3E). We then generated the *Mapk11* and *Hipk3* knockout mice by TALEN technology (see Materials and Methods section), respectively (Supplementary information, Figure S4A and S4B). Both types of mice were born in a roughly Mendelian ratio (Hipk3: 21% −/−, 23% +/+ and 56% +/−, from 111 mice; Mapk11: 25% −/−, 23% +/+ and 52% +/−, from 295 mice) and were viable and fertile and of normal appearance/weight/size/major tissue size and weight, and had no obvious health problems when kept under standard specific-pathogen-free conditions, consistent with previous reports^32,33^. We crossed them to a well-established HD KI mouse model expressing endogenous mHtt proteins with a 140Q repeat^34^. Heterozygous knockout of *Hipk3* or *Mapk11* significantly lowered full-length Htt levels *in vivo* in the striata of the HD mice (*Hdh*^Q140/Q7^, Figure 2C and 2D). Noticeably, heterozygous *Hipk3* knockout mice had lower Htt levels at 10 months of age but not at 5 months old (not shown), possibly because Hipk3 expression level is higher in HD only at later ages (Figure 3D).

To test whether Mapk11 or Hipk3-mediated Htt regulation is specific, we tested a number of control proteins including β-actin, Gapdh, Calnexin, Tuj1, Map2, α-tubulin and β-tubulin. None of their levels were significantly reduced in *Mapk11* or *Hipk3* knockout mice (Supplementary information, Figure S5A-S5D). Of other polyQ proteins including ataxin1, 2&3, we could only detect ataxin3 expression in our models and its levels were not reduced in the *Mapk11* or *Hipk3* knockout mice (Supplementary information, Figure S5A-S5D). Taken together, these results suggest that the effects of Mapk11 and Hipk3 on the mHtt levels are specific.

### Both MAPK11- and HIPK3-mediated effects on HTT levels are mHTT-dependent

Interestingly, we found that both the MAPK11- and HIPK3-mediated effects occurred only in HD, but not in wild-type (WT) cells/tissues (Figure 2). Two possibilities may account for this observation. First, the regulation of HTT by these kinases might be mHTT-specific, i.e., MAPK11 and HIPK3 only regulate mHTT but not wtHTT. Second, the regulation is mHTT-dependent, i.e., both mHTT and wtHTT levels are regulated by MAPK11/HIPK3 only in presence of mHTT. The second possibility seems to be the case, because both the wtHTT and mHTT levels appeared to be lowered similarly in the HD cells or tissues (Supplementary information, Figure S6A and S6B). To further clarify the mHTT-dependence, we expressed the mHTT-exon1 (Q72) fragment^35^ in WT cells (*STHdh*^Q7/Q7^) (Supplementary information, Figure S6C), in which the wtHtt level is insensitive to Mapk11 or Hipk3 (Figure 2A). The regulation of the wtHtt level by Mapk11 or Hipk3 was then enabled in the presence of mHTT-exon1 (Q72) but not wtHTT-exon1 (Q25) protein fragments (Figure 3A and 3B). This suggests that mHTT is sufficient to drive the Mapk11/Hipk3-mediated modulation of Htt that is absent in WT cells. This effect is dependent on mHTT, because expression of the long polyQ itself (Q72-GFP) or another polyQ protein (Atxn1 (Q92)) did not have such effects (Supplementary information, Figure S6D). Moreover, we specifically knocked down *mHtt* in the *STHdh*^Q7/Q111^ cells with an allele-specific siRNA that selectively lowers mHtt via targeting the CAG repeat region^36,37,38^, and the Mapk11/Hipk3-mediated modulation of both mHtt and wtHtt was diminished (Figure 3C); suggesting that mHTT is required for this regulation. Taken together, this indicates that MAPK11/HIPK3-mediated HTT modulation is dependent on the presence of mHTT proteins: in WT cells without mHTT or in HD cells in which *mHTT* is knocked down, MAPK11/HIPK3-mediated regulation of HTT is largely absent and thus the HTT level is insensitive to these kinases (Figures 2 and 3C); in HD cells with mHTT or in WT cells with exogenously expressed mHTT-exon1 fragments, the MAPK11-mediated and HIPK3-mediated HTT regulation is enabled, and both the mHTT and wtHTT levels become sensitive to these kinases (Figures 2, 3A and 3B).

Our observation of mHTT-dependent HTT modulation suggests that mHTT may enhance its own level by enabling positive regulation mediated by MAPK11 and HIPK3, which may contribute to disease progression.

### mHTT may confer positive effects on MAPK11 and HIPK3

Since mHTT is required for the MAPK11/HIPK3-mediated effects, we speculate that mHTT may elevate the levels or activities of MAPK11 or HIPK3, granting them the capability of regulating HTT levels. Indeed, mHTT has been shown to activate p38 MAPKs and c-Jun N-terminal kinases (JNKs)^39^. MAPK11 is a family member of the p38 MAPKs, which are activated by mHTT. The *HIPK3* mRNA level is elevated by JNKs^26^, which are also activated by mHTT. Thus, these previous results suggest that mHTT may activate MAPK11 kinase activity and elevate the *HIPK3* mRNA level, conferring upon them the ability to regulate HTT levels.

In accord with this, the endogenous *Hipk3* mRNA level was significantly higher in HD cells (*STHdh*^Q7/Q111^) than in WT cells (*STHdh*^Q7/Q7^) as determined by qRT-PCR (Figure 3D, left). A similar phenomenon was also observed *in vivo* in the striata of HD mice (*Hdh*^Q140/Q7^) compared to WT controls (*Hdh*^Q7/Q7^) at the age of 6-8 months (Figure 3D, right), in line with previous reports in human brain tissues, where the HIPK3 expression is ∼33% higher in HD patients versus controls^40^. Noticeably, this difference was not present at earlier ages (2-4 months) (Figure 3D, middle), suggesting that mHtt may increase the expression of Hipk3 as the disease progresses. To further establish the relationship between mHTT and Hipk3 levels, we knocked down *Htt* (both mHtt and wtHtt) in HD cells (*STHdh*^Q7/Q111^), or wtHtt in WT cells, and measured *Hipk3* mRNA levels. The *Hipk3* mRNA levels are significantly reduced by lowering Htt in HD cells but not by lowering wtHtt in WT cells (Figure 3E). This confirms the positive regulation of *Hipk3* mRNA level by mHtt but not wtHtt. A similar phenomenon was observed in patient iPSC-derived neurons (Figure 3F). We observed no increase of *Mapk11* mRNA levels in HD cells (*STHdh*^Q7/Q111^) compared to the WT cells (*STHdh*^Q7/Q7^) (Supplementary information, Figure S6E, left), and a marginal but insignificant increase in the striata of HD mice (*Hdh*^Q140/Q7^) compared to WT controls (*Hdh*^Q7/Q7^) (Supplementary information, Figure S6E, right), suggesting that mHtt could enhance kinase activity rather than the Mapk11 level. Consistent with this, over-expression of mHTT (HTTexon1 (Q72)) but not wtHTT (HTTexon1 (Q25)) increased the phosphorylation of MAPK11 detected by the phospho-p38 MAPK antibody (Figure 3G). MAPK11 was over-expressed in this experiment due to a lack of specific antibody-detecting endogenous phosphor-MAPK11. To confirm activation of endogenous MAPK11, we assessed its ability to phosphorylate Atf2^41^. We found that Atf2 phosphorylation was significantly reduced in the striata of *Mapk11* knockout mice (Figure 3H), confirming Atf2 as a Mapk11 substrate. Interestingly, Atf2 phosphorylation was significantly increased in the striata of HD mice (*Hdh*^Q140/Q7^) compared to WT controls (*Hdh*^Q7/Q7^) (Figure 3I), suggesting that Mapk11's kinase activity was enhanced in HD cells. The enhancement of MAPK11 kinase activity by mHtt is could be partially contributed by elevated oxidative stress, because lowering the oxidative stress by treatment of the NADPH oxidase inhibitor diphenyleneiodonium chloride (DPI)^42^ significantly decreased mHTT-induced MAPK11 phosphorylation (Figure 3G).

Collectively, our data reveal the positive feedback regulation of HTT mediated by MAPK11 or HIPK3. While this may not be the only mechanism for their mHTT-dependent effects, it suggests a mechanism through which mHTT enhances its own level and thus contributes to its own accumulation and thereby, disease progression.

### MAPK11- and HIPK3-mediated effects are dependent on their kinase activities

**Figure 4:**
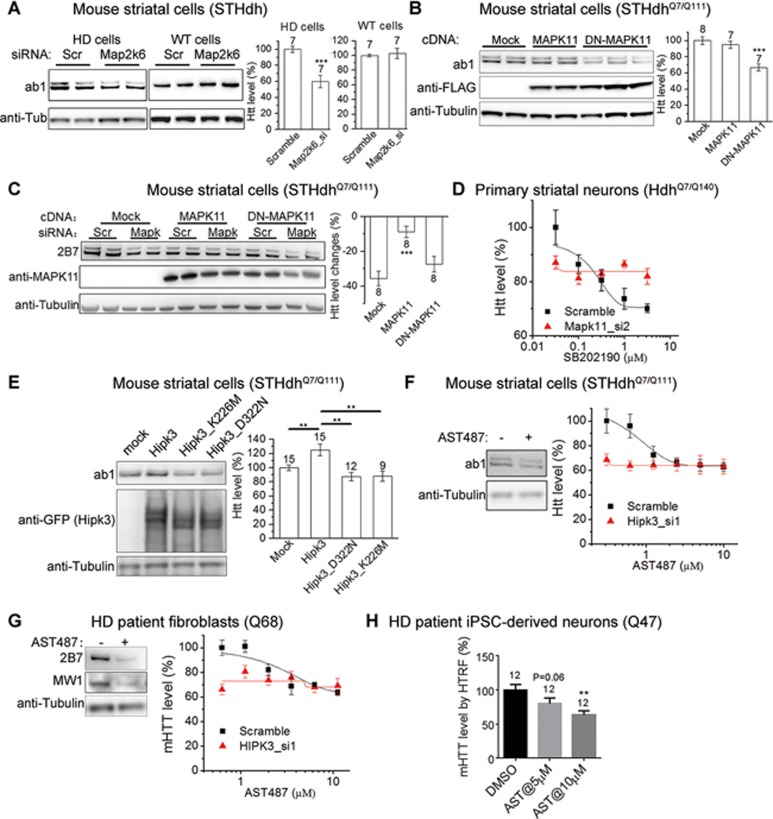
Mapk11 and Hipk3 regulate Htt levels via their kinase activities. **(A)** Representative western blots and quantifications of Htt levels in the HD (*STHdh*^Q7/Q111^) cells or WT (*STHdh*^Q7/Q7^) cells transfected with the indicated siRNAs. **(B)** Representative western blots and quantifications of Htt levels in the HD (*STHdh*^Q7/Q111^) transfected with dominant negative *MAPK11* cDNA (*DN-MAPK11*) versus *MAPK11* cDNA (*MAPK11*) plasmids. **(C)** Representative western blots and quantifications of Htt levels in the HD (*STHdh*^Q7/Q111^) transfected with indicated siRNAs targeting *Mapk11* and cDNA plasmids expressing MAPK11 or DN-MAPK11. **(D)** In primary cultured striatal neurons from *Hdh*^Q7/Q140^ mice transfected with the scramble control siRNA or the *Mapk11* siRNA (Mapk11_si2), the dose-dependent curves of the Htt levels upon treatment of the p38 Mapk inhibitor SB202190 were measured by HTRF using the 2B7/2166 antibody pair. Data plotted as mean and SEM, *n* = 6. Curves were fitted by the Sigmoidal dose-dependent curve. **(E)** Representative western blots and quantifications showing that over-expression of the wild-type *Hipk3* cDNA (HIPK3) increases Htt levels in the *STHdh*^Q7/Q111^ cells, while the kinase-dead Hipk3 (K226M and D322N) has no such effects. **(F)** Left, Representative western blots showing that treatment with the Hipk3 inhibitor AST487 reduces Htt levels in *STHdh*^Q7/Q111^ cells at 10 μM. Right, in the *STHdh*^Q7/Q111^ cells transfected with the scramble control siRNA or the *Hipk3* siRNA (Hipk3_si1), the dose-dependent curves of the Htt levels upon treatment of AST487 were measured by HTRF using the 2B7/2166 antibody pair. Transfection of the *Hipk3* siRNA abolished the Htt lowering effect of AST487. Data plotted as mean and SEM, *n* = 10. Curves were fitted by the Sigmoidal dose-dependent curve. **(G)** Similar to **(F)**, but in immortalized HD patient fibroblasts (Q68). In the HTRF experiments in the right panel, HD Q68 fibroblasts were transfected with the scramble control siRNA or the *HIPK3* siRNA (HIPK3_si1), and the mHTT levels were measured by HTRF using the 2B7/MW1 antibody pair. Transfection of the *HIPK3* siRNA abolished the effect of AST487. Data plotted as mean and SEM, *n* = 10. Curves were fitted by the Sigmoidal dose-dependent curve. **(H)** In the HD patient iPSC-derived neurons (Q47), mHTT levels upon treatment of AST487 were measured by HTRF using the 2B7/MW1 antibody pair. Data plotted as mean and SEM, *n* = 12. The statistical analysis was performed by one-way ANOVA and Dunnett's *post hoc* tests. ^**^P < 0.01.

In order to lower mHTT levels and treat HD via targeting MAPK11 or HIPK3, we asked whether their effects on mHTT levels were dependent on their kinase activities, which could be targeted by low-molecular weight compounds. MAPK11 belongs to the p38 MAPK family, and it is phosphorylated by MAP2K6 to activate its kinase activity^25^. In HD cells including both *STHdh*^Q7/Q111^ and iPSC-derived Q47 neurons, knockdown of *MAP2K6* by siRNAs (Supplementary information, Figure S2C) also reduced mHTT levels, an effect not seen in WT cells (Figures 2B and 4A). In addition, the MAP2K6-mediated effect on HTT is dependent on MAPK11 (Supplementary information, Figure S3A). Thus, MAPK11's effect on HTT is likely to be dependent on its kinase activity, which is activated by MAP2K6. Consistent with this idea, we found that dominant negative MAPK11 (DN-MAPK11)^43^ reduced Htt levels when over-expressed in HD cells (Figure 4B). Moreover, over-expression of MAPK11 but not DN-MAPK11 significantly suppressed the Htt lowering effect of *Mapk11* knockdown (Figure 4C), confirming that MAPK11's kinase activity is required for its role in regulating HTT levels. We then tested the potential effects of the Mapk11 inhibitor SB202190^44^ on Htt levels. However, SB202190 treatment failed to reduce Htt levels in *STHdh*^Q7/Q111^ (not shown), possibly because of the off-target effects on other kinases. We therefore tested its effect in other HD models and discovered that SB202190 treatment was able to reduce Htt levels in a Mapk11-dependent manner in primary striatal neurons cultured from the KI HD mice (*Hdh*^Q7/Q140^) (Figure 4D), further confirming the kinase-activity dependence and providing an entry point for HD drug discovery.

Similarly, over-expression of mouse *Hipk3* cDNA increased endogenous Htt levels, while over-expression of the kinase-dead *Hipk3* cDNAs (K226M and D322N)^45,46^ gave a modest reduction in Htt (Figure 4E). This suggests that Hipk3-mediated Htt regulation is also kinase activity-dependent. Consistently, the HIPK3 kinase inhibitor AST487^47^ effectively lowers mHTT levels (Figure 4F and 4G), and this effect is mitigated by knocking down *HIPK3* (Figure 4F and 4G, right panels), confirming that AST487 reduces mHTT levels via inhibiting HIPK3. Finally, AST487 also reduced mHTT levels in the HD patient iPSC-derived neurons (Figure 4H), confirming the effects in a human neuronal model expressing a polyQ length that is close to the ones commonly found in HD patients.

In summary, MAPK11 and HIPK3 regulate HTT through their kinase activities, opening up possibilities for HD drug development.

### MAPK11 regulates HTT mRNA stability while HIPK3 regulates HTT degradation via autophagy

**Figure 5:**
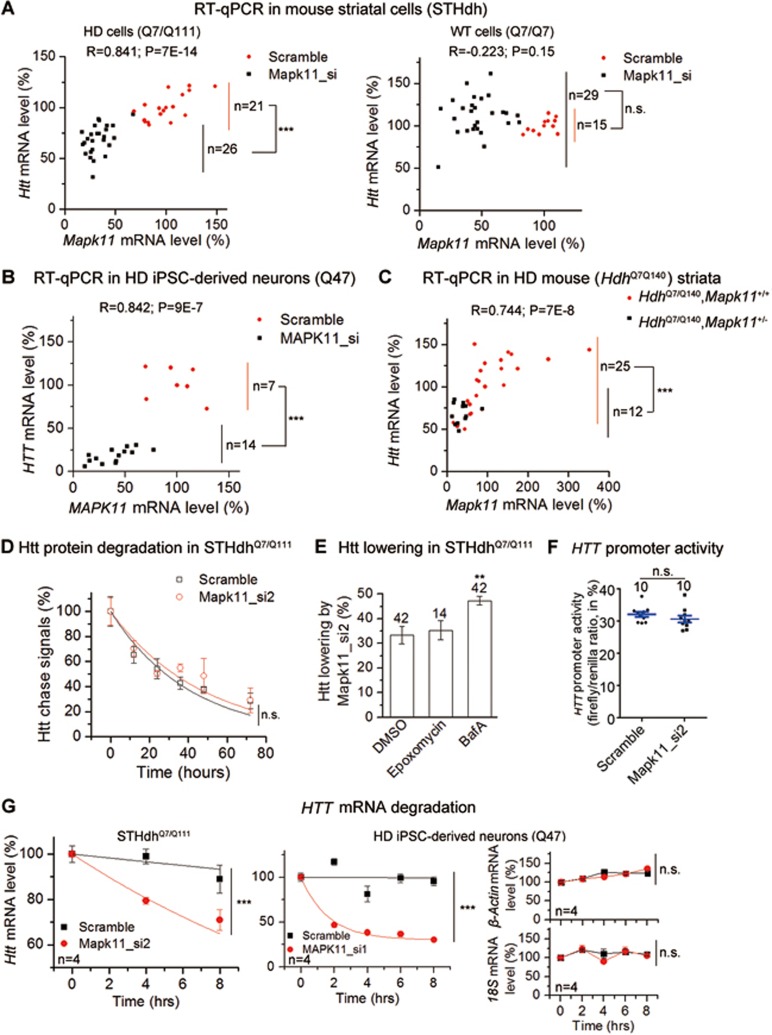
Mapk11 regulates *HTT* mRNA levels via regulation of *HTT* mRNA stability. **(A)** RT-qPCR results in *STHdh* cells showing that knockdown of *Mapk11* lowers *Htt* mRNA levels in HD (left) but not wild-type cells (right). The *X*-axis indicates the *Mapk11* mRNA expression level, whereas the *Y*-axis indicates the *Htt* mRNA level. As for **B**-**D**. Each single point exhibits the *Htt* versus *Mapk11* mRNA levels of an independently transfected well, and the numbers are indicated (*n*). All data were normalized to the average of scrambled siRNA-transfected control samples. Red, scrambled siRNA-transfected control samples; Dark, cells transfected with either of the two *Mapk11* siRNAs (Mapk11_si1&Mapk11_si2). The correlation coefficient *R* and *P* values on top of each plot were calculated by Spearman correlation analysis. “^***^” on the right side of each plot indicates that *P*< 0.001 calculated by unpaired two-tailed *t* tests for the *Htt* mRNA levels in *Mapk11* siRNA-transfected sample versus the control. **(B)** Similar to **(A)**, but in HD patient iPSC-derived neurons (Q47). Data plots and statistical analysis were performed in the same way as in **(A)**. **(C)** Similar to **(A)**, but the mRNAs were extracted from *in vivo* mouse striata (*Hdh*^Q7/Q140^ with or without heterozygous knockout of *Mapk11*, 4-6.5-month-old), and the statistical analyses were performed the same. The “*n*” numbers indicate the number of mice. **(D)** The non-radioactive pulse-chase experiment showing that knockdown of *Mapk11* does not influence Htt protein degradation. The siRNAs were transfected in *STHdh*^Q7/Q111^ cells. Three independently transfected wells for each point were tested. Data plotted as mean and SEM. The statistical analysis was performed by two-way ANOVA to determine if the transfection influences the protein degradation. “ns” on the right side of the plot indicates no statistically significant interaction between siRNA transfection and Htt protein degradation. **(E)** The Htt lowering by *Mapk11* siRNA (Mapk11_si2) was measured in *STHdh* cells by 2B7/2166 HTRF. The proteasome inhibitor epoxomycin or the autophagy inhibitor bafilomycin A (BafA) did not reduce the lowering effect given by *Mapk11* knockdown. Bars represent mean and SEM, and the numbers indicate the number of independently transfected wells. The statistical analysis was performed by one-way ANOVA and Dunnett's *post hoc* tests. ^**^P< 0.01. **(F)** The *Htt* promoter activity was measured in *STHdh*^Q7/Q111^ by the luciferase reporter assay (see Materials and Methods). Knockdown of *Mapk11* did not significantly influence the *Htt* promoter activity. Blue lines represent mean and SEM, and 10 independently transfected wells were tested for each condition. The statistical analysis was performed by unpaired two-tailed *t* tests. **(G)** *Htt* mRNA stability measurements in *STHdh*^Q7/Q111^ cells (left) and HD patient iPSC-derived neurons Q47 (right). The cells were transfected with indicated siRNAs for 36 h and then treated with actinomycin D to stop new mRNA synthesis. The *Htt/HTT* mRNA level was then measured at different time points using RT-qPCR, normalized to *Hprt/HPRT* mRNA levels and plotted as percentage of the initial time point. For HD patient iPSC-derived neurons, β-actin and 18S mRNA were used as controls, which were not influenced by *MAPK11* knockdown. Since their degradation is close to HPRT and their levels were normalized to HPRT at each time point, the normalized levels were relatively flat across time points. Data were plotted as mean and SEM. The statistical analysis was performed by two-way ANOVA to determine if the transfection influences the mRNA degradation. ^***^P< 0.0001 for the effect of *MAPK11* siRNA on *Htt* mRNA degradation.

In mouse striatal HD cells (*STHdh*^Q7/Q111^) but not WT cells (*STHdh*^Q7/Q7^), *Htt* mRNA was significantly reduced by knocking down *Mapk11* (Figure 5A, statistics on right), and this showed a significant positive correlation with *Mapk11* mRNA levels (Figure 5A, statistics on top). Similar phenomena were observed in HD patient iPSC-derived Q47 neurons and HD mouse (*Hdh*^Q7/Q140^) striata (Figure 5B and 5C), suggesting that MAPK11 positively regulates *HTT* mRNA levels. Noticeably, the degree of *HTT* mRNA lowering somewhat differs from the reduction of HTT protein levels (Figure 2 versus Figure 5). This is likely because the HTT protein level is determined by the balance point of protein synthesis and degradation, which is influenced by not only *HTT* mRNA levels, but also the dynamics of HTT translation and degradation. This will result in a non-linear relationship between mRNA and protein levels.

Consistent with the observation that MAPK11 may regulate HTT protein levels through regulating *HTT* mRNA levels, the Htt protein degradation rate was not increased by knocking down *Mapk11* (Figure 5D). In addition, Mapk11-mediated Htt regulation was not affected by inhibitors of proteasome or autophagy (Figure 5E), the two major protein degradation machineries.

There are two major mechanisms through which MAPK11 may regulate *HTT* mRNA levels: transcription and mRNA stability. To test the possible regulation of *HTT* transcription by MAPK11, we examined *HTT* promoter activity using a previously published reporter assay for the *HTT* promoter^48^ and utilizing the longest version of the described *HTT* promoter. *Mapk11* knockdown did not change *HTT* promoter activity in *STHdh*^Q7/Q111^ cells (Figure 5F), suggesting that the Mapk11-mediated effect on *HTT* mRNA is not mediated by regulation of *HTT* transcription, but probably through *HTT* mRNA stability. We therefore measured *HTT* mRNA degradation and discovered that it was significantly accelerated by knocking down *MAPK11* in both *STHdh*^Q7/Q111^ cells and patient iPSC-derived HD Q47 neurons (Figure 5G), confirming that MAPK11 regulates *HTT* mRNA stability.

In contrast, knockdown of *Hipk3* did not significantly influence *Htt* mRNA levels (Supplementary information, Figure S7A). On the other hand, *HIPK3* knockdown accelerated HTT protein degradation (Supplementary information, Figure S7B and S7C). In addition, the *HIPK3* knockdown-induced mHTT reduction was blocked by the autophagy flux inhibitors NH_4_Cl and bafilomycin A, but not by the proteasome inhibitors epoxomycin and MG132 (Supplementary information, Figure S7D), suggesting that HIPK3 may regulate mHTT levels via autophagy. To further confirm the involvement of autophagy, we knocked down several essential autophagy genes (*ATG5*, *ATG12*, *ATG16L*, *SQSTM1*) and found that the *HIPK3* knockdown-induced lowering of mHTT was subsequently lessened (Supplementary information, Figure S7E). Taken together, this indicates that MAPK11 regulates HTT at the mRNA level, whereas HIPK3 regulates mHTT, at least partially, through autophagy-mediated mHTT degradation.

**Figure 6:**
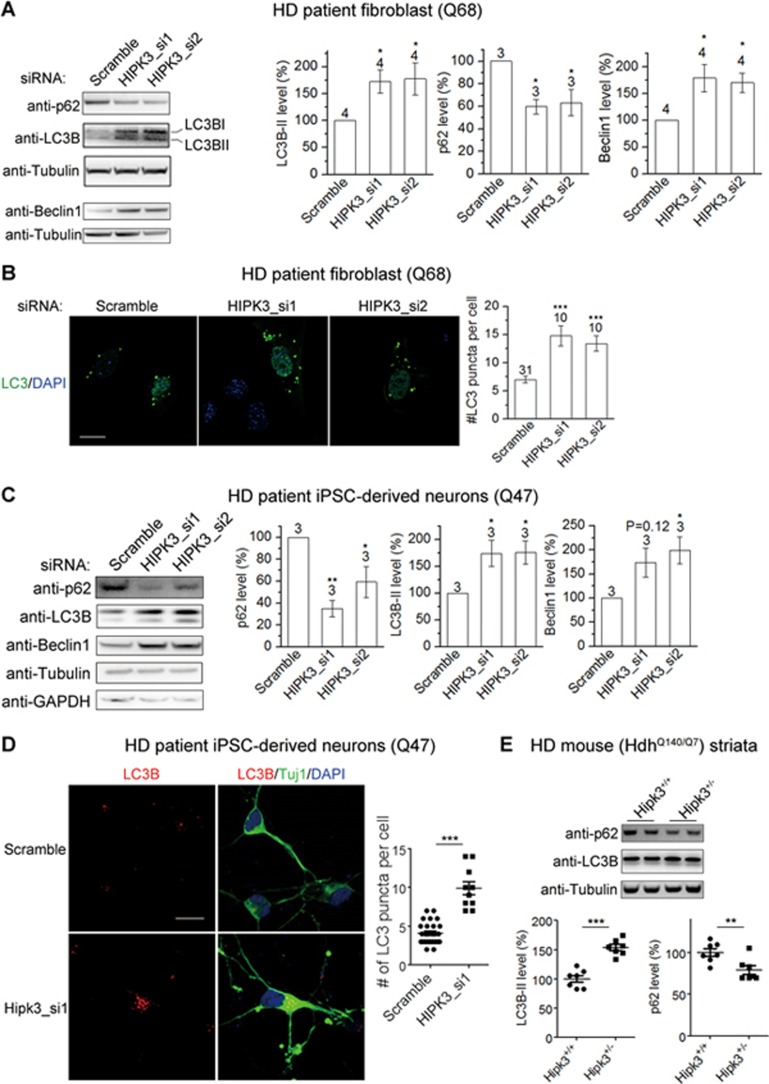
Hipk3 regulates autophagy flux. **(A)** Representative western blots and quantification of human patient fibroblasts (Q68) transfected with *HIPK3* siRNAs versus scrambled control siRNA. LC3B-II and Beclin1 levels were increased whereas the p62 levels were decreased. The statistical analysis was performed by one-way ANOVA and Dunnett's *post hoc* tests. ^*^P < 0.05. **(B)** Representative images and quantification of microscopy experiments showing that the number of GFP-LC3 puncta was increased by knocking down *HIPK3* in patient fibroblasts (Q68) cells transfected with the GFP-LC3 plasmid. Scale bar, 20 μm. The statistical analysis was performed by one-way ANOVA and Dunnett's *post hoc* tests. ^***^P < 0.001. **(C)** Similar to **(A)** but from patient iPSC-derived neurons (Q47). The statistical analysis was performed by one-way ANOVA and Dunnett's *post hoc* tests. ^*^P < 0.05; ^**^P < 0.01. **(D)** Representative images and quantification of endogenous LC3II puncta in patient iPSC-derived neurons (Q47) by immunostaining with the LC3II antibody. Over-expression of LC3 led to toxicity in these cells and changed their morphology, and thus endogenous LC3II puncta were examined. Scale bar, 20 μm. The statistical analysis was performed by two-way unpaired *t* tests. ^***^P < 0.001. **(E)** Representative western blots and quantification of mouse striata tissue lysate samples. The genotypes have been indicated. Each point represents a single mouse. Note that the samples were from the same mice used in Figure 2D. For all bar plots, data represent mean and SEM, and the numbers indicate biological replicates for western blots, or the number of cells in imaging analysis. Images were analyzed blindly by ImageJ.

To further investigate the potential effect of HIPK3 on autophagy, we tested the effect of *HIPK3* knockdown on autophagy markers p62 (encoded by *SQSTM1*), LC3 and Beclin1. Autophagosome-bound lipidated LC3-II has been widely used as an indicator of autophagosomes^49^. Beclin1 is a critical subunit of a class III PI-3 kinase (Vps34) complex required for autophagosome biogenesis^50^. The p62 protein is a receptor that binds LC3-II and ubiquitin, and is degraded when the autophagosome fuses with the lysosome^51^. p62 is a preferred marker for measuring autophagic flux as its decreased levels correlate with increased flux^49^. *HIPK3* knockdown led to increased LC3-II and Beclin1 levels and decreased p62 levels in HD patient fibroblasts (Q68), suggesting an increase of autophagosomes and autophagic flux facilitates mHTT degradation (Figure 6A). To further confirm the effect of HIPK3 on autophagy, we expressed GFP-LC3B and observed an increase in the number of LC3 puncta that mark autophagosomes (Figure 6B), suggesting increased autophagy. A similar phenomenon was observed in the patient iPSC-derived Q47 neurons (Figure 6C and 6D), confirming the effect in a human neuronal model with a polyQ length that is close to the ones commonly found in HD patients. Endogenous LC3 was assessed in these neurons by immunostaining using an antibody that preferentially detects LC3II^52^, because over-expression of *LC3-GFP* or other *LC3* cDNA led to morphological changes and cytotoxicity in these neurons (not shown). Finally, the effect was also confirmed *in vivo* in heterozygous *Hipk3* knockout HD mice (Figure 6E).

**Figure 7:**
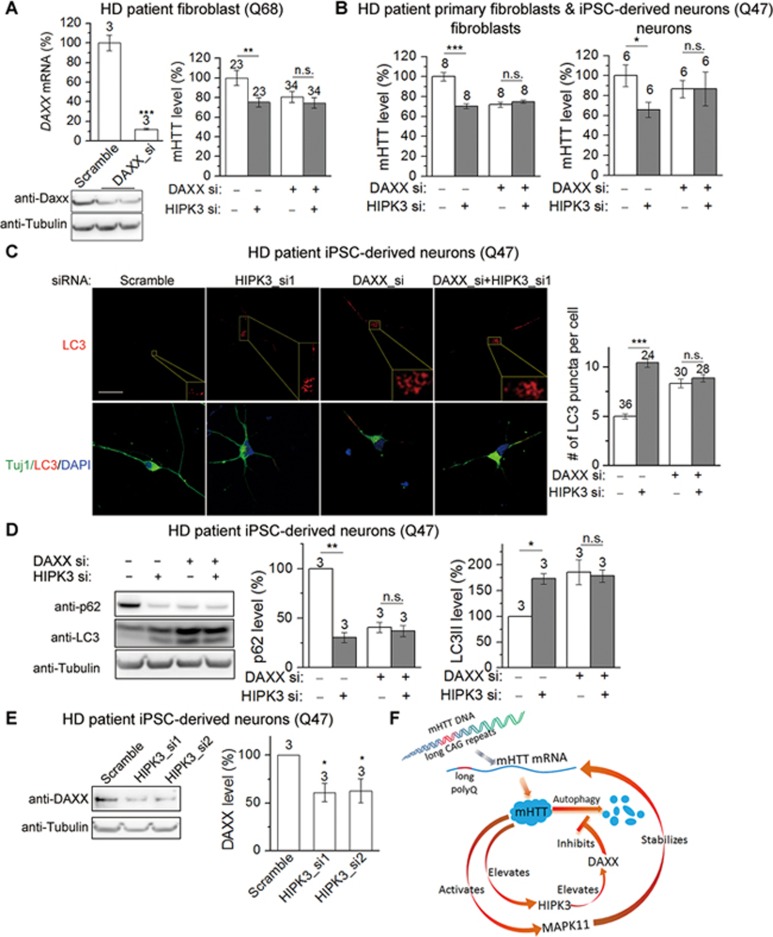
Hipk3 regulates mHtt levels via DAXX. **(A)** Left, RT-qPCR and western blots confirming the knockdown of *DAXX* in HD patient fibroblasts (Q68) by its siRNA. Right, mHTT levels in HD patient fibroblasts (Q68) measured by HTRF using the antibody pair 2B7/MW1, after transfection of the indicated siRNAs. Transfection of the *DAXX* siRNA blocked the effect of knocking down *HIPK3*. **(B)** mHTT levels in HD patient (Q47) primary fibroblasts (left) or HD patient iPSC-derived neurons (right) measured by HTRF using the antibody pair 2B7/MW1, after transfection of the indicated siRNAs. Transfection of the *DAXX* siRNA blocked the effect of knocking down *HIPK3*. **(C)** Representative images and quantification of endogenous LC3II puncta in patient iPSC-derived neurons (Q47) transfected with the indicated siRNAs. Scale bar, 20 μm. The quantification of the puncta was performed blindly, the boxed regions have been magnified and exhibited at the lower right corner for better view of the puncta, but the counting was not limited to the boxed regions. The statistical analysis was performed by two-way unpaired *t* tests. **(D)** Representative western blots and quantification of p62 and LC3 blots of patient iPSC-derived neurons (Q47) transfected with the indicated siRNAs. **(E)** Representative western blots and quantification of DAXX in patient iPSC-derived neurons (Q47) transfected with the indicated siRNAs. **(F)** A model illustrating the positive feedback loop mediating MAPK11 and HIPK3's effect on mHTT levels. For all bar plots, data represent mean and SEM. The statistical analysis was performed by two-way unpaired *t* tests for **A** to **D**, and by one-way ANOVA followed by Dunnett's *post hoc* tests for **E** and **F**. ^*^P < 0.05; ^**^P < 0.01; ^***^P < 0.001; ns, *P* > 0.05.

As a major substrate of HIPK3, DAXX directly interacts with and is phosphorylated by HIPK3^46,53^ and suppresses autophagy flux^54^. Thus, DAXX may participate in the HIPK3-mediated regulation of mHTT levels via autophagy. Knocking down *DAXX* (Figure 7A) blunts the effect of HIPK3 on regulation of mHTT levels in patient fibroblasts and patient iPSC-derived neurons (Figure 7A and 7B), suggesting the involvement of DAXX in HIPK3-mediated mHTT regulation.

We then further tested if HIPK3 regulates autophagy through DAXX as predicted. In patient iPSC-derived neurons (Q47), the knockdown of *DAXX* mitigated the effect of *HIPK3* knockdown on LC3 puncta and LC3II/p62 levels (Figure 7C and 7D). This confirms that HIPK3 regulates autophagy via DAXX. Finally, lowering HIPK3 significantly reduced the level of DAXX protein (Figure 7E), possibly due to a reduction of phosphorylated DAXX protein. Taken together, this indicates that HIPK3 regulates mHTT levels via the autophagy suppressor DAXX.

In summary, MAPK11 and HIPK3 regulate mHTT levels through a potential positive feedback loop via distinct mechanisms, as illustrated in the model in Figure 7F.

### Lowering MAPK11 rescues HD-relevant phenotypes in an HD KI mouse model

In order to validate the therapeutic significance of the discovered modifiers of mHTT levels, it is critical to test whether these targets influence HD-relevant phenotypes. *Hipk3* knockout may cause metabolic defects^32^, while the *Mapk11* knockout does not seem to have obvious phenotypes based on previous reports^33^. While the previously reported models were generated by different approaches than ours, all available information suggests that targeting Mapk11 is probably safer. Thus, we prioritized Mapk11 for testing the potential rescue of HD-relevant phenotypes.

**Figure 8:**
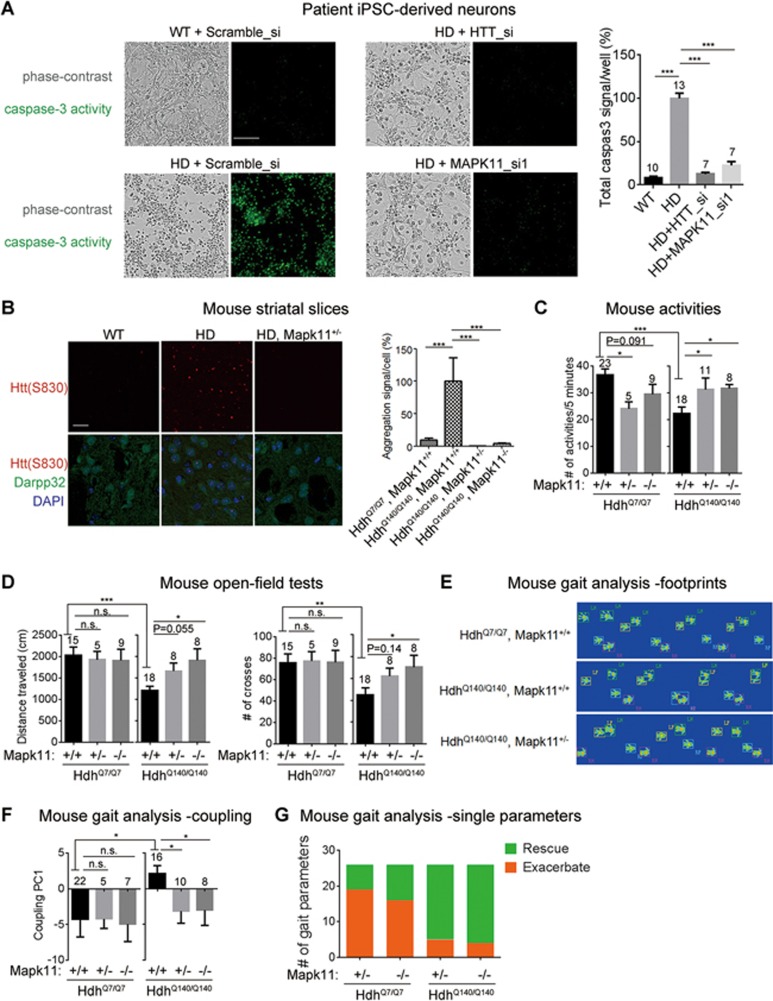
Lowering Mapk11 rescues HD-relevant phenotypes in iPSC-derived HD neurons and HD knockin mouse model. **(A)** Left panels, representative phase-contrast (for cell confluence and morphology) and green fluorescence images (for caspase-3 activity using the caspase-3 detection dye) of the wild-type (WT, Q19) or the Huntington's disease (HD, Q47) iPSC-derived neurons transfected with the indicated siRNAs under BDNF deprived conditions for 48 h. Scale bar, 100 μm; Right panel, quantification of the number of caspase-3 signals (green particle counts × size × mean intensity) per field by IncuCyte software. The numbers on top indicate the number of independently transfected wells. 4 fields of each well were imaged for quantification by IncuCyte inside the cell culture incubator. All the signals were normalized to the average of the HD cells. Bars represent mean and SEM. The statistical analysis was performed by one-way ANOVA and Dunnett's *post hoc* tests. **(B)** Left panels, representative images of the Htt aggregates (by the antibody S830) alone or merged with DAPI and Darpp32. The scatter plot, quantification of the total aggregate signals (aggregate counts × size × mean intensity) per cell by particle analysis using ImageJ. Data were from 12 different slices from 3 mice of each genotype. Scale bar, 20 μm. The imaging and the analysis were performed blindly before revealing the genotypes. The statistical analysis was performed by one-way ANOVA and Dunnett's *post hoc* tests. **(C)** The total number of active behaviors (activities) in 5 min for each mouse of the indicated genotypes tested in the pen holder with mashed surface. Unpaired two-tailed *t* test was performed between the HD (*Hdh*^Q140/Q140^) and WT (*Hdh*^Q7/Q7^) mice without *Mapk11* deletion (*Mapk11*^+/+^); one-way ANOVA and Dunnett's *post hoc* tests were performed within the HD and WT groups. **(D)** Left, the total distance traveled in 15 min for mice of the indicated genotypes tested in the open-field. Right, the open-field was divided into 4 × 4 virtue grids for analysis, and the number of times that each mouse crossed the borders of the middle 4 blocks was quantified. The statistical analysis was performed and indicated as in **(C)**. **(E)** Representative footprints of mice of indicated genotypes. **(F)** The plot of the top principle component (Coupling PC1) of the gait coupling parameters (Supplementary information, Table S1). The statistical analysis was performed and indicated as in **(C)**. **(G)** Summary of the number of rescued (green) versus exacerbated (orange) parameters by *Mapk11* heterozygous or heterozygous knockout. The category was judged by the direction of changes. Heterozygous or homozygous knockout of *MAPK11* in HD mice led to predominantly more rescues (green) than exacerbations (orange), suggesting rescue effects. See Supplementary information, Table S2 for the selected parameters. For all plots, error bars represent mean and SEM. The numbers on top of each plot indicate the number of mice. For all the behavioral experiments and aggregation immunofluorescence, mice were tested blindly and their genotypes were revealed after data recording and analysis. Immunofluorescence data were obtained from 11 month old mice and behavioral data were obtained from 7.5 month old mice.

At the cellular level, it has been reported that mHTT toxicity induces caspase-3 activation under the BDNF-deprived culture condition in HD patient iPSC-derived neurons. Thus caspase-3 activation could be used as a readout for mHTT-mediated toxicity^55^. We found that *MAPK11* knockdown significantly lowered the mHTT-dependent caspase-3 activation under this condition, suggesting that lowering MAPK11 ameliorates mHTT toxicity in HD patient iPSC-derived neurons (Figure 8A).

mHTT forms insoluble aggregates in the striata of aged HD mice, and these aggregates can be recognized by immunofluorescence (Figure 8B; from 11-month-old homozygous HD mice *Hdh*^Q140/Q140^). Compared to homozygous mice, heterozygous HD mice *Hdh*^Q7/Q140^ exhibited many fewer aggregates, and so we focused on homozygous HD mice to better quantify protein aggregates and assess Mapk11's effect on mHtt levels. Remarkably, aggregation levels in these homozygous HD mice were reduced to wild-type levels when the mice were also either *Mapk11*^+/−^ or *Mapk11*^−/−^ (Figure 8B). This is quite striking considering that soluble mHTT was reduced by less than 50% in the *Mapk11*^+/−^ condition (Figure 2D; Supplementary information, Figure S8B). Meanwhile, since *Mapk11*^+/−^ resulted in mHtt reduction well before the formation of aggregates, it is likely that either the *Mapk11*^+/−^ or *Mapk11*^−/−^ genotype reduced soluble mHtt at early ages and prevented formation of mHtt aggregates (Figure 8B). This is consistent with previous studies, which showed a drastic reduction of mHtt aggregates by short-term Htt-targeting using siRNA or ASO treatment^8,9^.

We further investigated whether the heterozygous or homozygous knockout of *Mapk11* also rescues HD-relevant behavioral phenotypes in mice. We explored a number of behavioral assays including the rotarod test, gripping force measurement, the open-field test, activity measurement, the water maze test and the nest building test in both HD and WT mice. Consistent with other reports^34^, we observed several motor function-related phenotypes in this model. At the age of 7.5 months, the homozygous HD (*Hdh*^Q140/Q140^) mice showed less activity (measured by the frequency of active behaviors including rearing and climbing) in a pen holder with mashed surface (Figure 8C, comparison between the first group of each plot). They also exhibited mobility defects in the open-field test (Figure 8D, comparison between the first group of each plot). However, we failed to detect reliable behavioral phenotypes in the heterozygous HD mice (*Hdh*^Q7/Q140^, Supplementary information, Figure S8A) and thus performed further testing in the homozygous HD mice. By crossing the *Mapk11* knockouts to the HD mice for several generations, we obtained both *Mapk11* heterozygous (*Mapk11*^+/−^) and homozygous (*Mapk11*^−/−^) knockouts and related controls (*Mapk11*^+/+^) in both the HD (*Hdh*^Q140/Q140^) and WT (*Hdh*^Q7/Q7^) backgrounds. The soluble mHtt level was significantly lowered by *Mapk11*^+/−^ in striata from these mice (Supplementary information, Figure S8B), similar to heterozygous HD mice (Figure 2D). Both *Mapk11*^+/−^ and *Mapk11*^−/−^ genotypes showed significant (*P* < 0.05) or marginal (*P* = 0.1) rescue of the behavioral defects of the HD mice in activity (Figure 8C) and open-field tests (Figure 8D). These data confirm the protective effect of lowering Mapk11 on HD phenotypes. Of note, although *Mapk11*^+/−^ and *Mapk11*^−/−^ caused a mild reduction of the activity frequency in the WT background, such an effect was reversed in the HD background (Figure 8C). In the open-field tests, *Mapk11*^+/−^ and *Mapk11*^−/−^ did not have positive effects in the WT background, but they did show a rescue effect in the HD background (Figure 8D). The results confirm that the effects of *Mapk11*^+/−^ and *Mapk11*^−/−^ on motor functions are HD-specific since no positive effects were observed in the WT background.

HD patients have gait phenotypes and show impaired coupling^56^. We thus further tested the potential gait phenotype of these HD mice by the gait analysis system, which records the gait of mice passing through a scoring lane (Supplementary information, Movies S1-S2). The HD mice showed some gait abnormalities with more irregular steps and more overlaps of footprints; these abnormalities seemed to be rescued in the *Mapk11*^+/−^ and *Mapk11*^−/−^ backgrounds (Figure 8E; Supplementary information, Movies S1-S2). Of the 214 parameters quantified by the gait analysis equipment, 36 are coupling-related parameters. In order to capture the major components of the gait coupling phenotype, we have applied principle component analysis^57^ to all these 36 coupling-related parameters and revealed the top principle component that depicts the defects in the HD mice (Figure 8F and Supplementary information, Table S1). Based on the PCA analysis, the gait defects were markedly improved in *Mapk11*^+/−^ and *Mapk11*^−/−^ HD mice (Figure 8F).

To further confirm Mapk11's effect, we analyzed all the 214 single parameters without PCA transformation. We identified 6 out of the 214 parameters that showed a significant (*P* < 0.05) difference between HD and WT animals (Supplementary information, Table S2, yellow blocks). None of these six parameters are standard parameters in describing gait defects and they are unlikely to capture the major differences between HD and WT sufficiently. Given that there are so many parameters analyzed, these six parameters may have just passed the *P*< 0.05 threshold by chance. To capture the gait defects more accurately, we selected all the 26 parameters that showed at least a 10% difference with a relatively small *P* value (*P* < 0.2, by unpaired two-tailed *t* tests) between the HD and WT groups. We then determined whether the phenotype captured by a single parameter was rescued or exacerbated by comparing the averages of *Mapk11*^+/−^ or *Mapk11*^−/−^ with *Mapk11*^+/+^ mice in the same Htt background; the judgment was solely based on the direction of the changes (Supplementary information, Table S2). If the parameters were not influenced, random noise should result in equal probability of rescue versus exacerbation. In the HD mice (Supplementary information, Table S2, right parts), *Mapk11*^+/−^ and *Mapk11*^−/−^ showed overwhelmingly more rescues (green) than exacerbations (orange), an outcome highly unlikely to have resulted from random noise (*Mapk11*^+/−^: 21 rescues versus 5 exacerbations, *P* = 0.0009; *Mapk11*^−/−^: 22 rescues versus 4 exacerbations, *P* = 0.00009; *Mapk11*^+/−^ & *Mapk11*^−/−^ together: 43 rescues versus 9 exacerbations, *P* = 5E−7). In the WT mice, *Mapk11*^+/−^ and *Mapk11*^−/−^ did not have such effects (Supplementary information, Table S2, left parts). These distributions were summarized in a bar plot (Figure 8G) and strongly suggested that lowering Mapk11 significantly rescued mHTT-induced gait phenotypes. Finally, while behavior phenotypes in the heterozygous HD mice (*Hdh*^Q7/Q140^) were too weak to be assayed (Supplementary information, Figure S8A), they exhibited significantly lower levels of the medium spiny neuron marker Darpp32 compared to the WT (Supplementary information, Figure S8C). Moreover, this phenotype was also rescued by heterozygous knockout of *Mapk11* (*Hdh*^Q7/Q140^, *Mapk11*^+/−^) (Supplementary information, Figure S8C), suggesting that the rescue effect of lowering Mapk11 is also present in heterozygous HD mice.

Collectively, our data provide encouraging *in vivo* evidence to establish Mapk11 as a potential therapeutic target for HD drug discovery. While we cannot entirely exclude the involvement of other mechanisms in the phenotypic rescue, lowering mHtt is most likely the major mechanism that rescues HD phenotypes, because lowering Mapk11 reduces mHtt *in vivo* and reduction of mHtt by various approaches has been shown to rescue HD phenotypes.

## Discussion

### Screening for HTT protein modifiers

In summary, using an unbiased genetic screen and validating the output, we have identified MAPK11 and HIPK3 as robust modulators of HTT levels both in cells and *in vivo* (Figure 2). This regulation is dependent on their kinase activity, providing promising opportunities for drug development (Figure 4). Finally, heterozygous or homozygous deletion of Mapk11 lowers mHTT aggregates in HD striata and rescues the behavioral phenotypes of the HD mice *in vivo*, confirming the therapeutic potential of reducing the activity of this protein kinase.

Disease progression-modifying treatments are highly desired and yet unavailable for neurodegenerative disorders. Lowering disease protein levels is a promising strategy to achieve this goal. For HD, previous screening efforts focused on protein aggregation, cleavage or toxicity^58,59,60,61^. Our present study focuses on decreasing soluble mHTT levels for two reasons: first, soluble mHTT has been shown to be closely related with toxicity^11,12,13^; second, as the mechanism underlying mHTT toxicity is unclear, we chose to focus on the presence of soluble mHTT as an earlier event in disease pathogenesis. This approach would ensure that changes achieved in protein level should cascade through downstream steps and impact disease-relevant phenotypes, including aggregation (Figure 8B).

In our previous screening effort using *Drosophila* S2R cells, we only obtained hits that upregulate mHTT when knocked down^30^. This is likely because the primary screen was performed in cells over-expressing exogenous mHTT fragments, and the screening system may elevate pathways that clear the over-expressed mHTT fragments and thus could be biased towards enhancer hits in a knockdown screen. Here we optimized the screen in human patient fibroblasts expressing the endogenous full-length human mHTT protein and focusing upon targets that may reduce mHTT when knocked down.

Other than MAPK11 and HIPK3, we identified a number of other targets (including the gene for MAPK11's upstream regulator *MAP2K6*) that reduced mHTT and rescued hESC-derived Q73 neurons in our knockdown screen (Figure 1). The negative correlation between mHTT-exon1 protein level and neuronal survival strongly suggests that these targets reduce mHTT toxicity through lowering its level (Figure 1E), although further validation with more replicates and in more HD models is definitely needed. MAPK11 and HIPK3, as well as the other newly identified targets have not previously been reported to have physical interactions with HTT. Thus, they probably regulate mHTT through indirect mechanisms rather than through a direct interaction with mHTT. Noticeably, besides the identified kinases, the targets USP39, RNF145 and PRPF19 are all related with ubiquitination processes, whereas MMP25, NPEPL1 and ECE1 are proteases/peptidases. This suggest that ubiquitination and protein cleavage likely play important roles in mHTT modulation. Like any other high-throughput screen, our screen may identify false-targets due to the limitations of cellular models, HTT detection and insufficient knockdown. The siRNA library used for our screens were ordered from Qiagen Inc., and most of them have been validated based on the manufacturer's information. Consistently, most of the siRNAs knocked down their targets more than 70% based on our qPCR validation of transcript levels in our present and previous studies. In addition, we tested four siRNAs targeting each gene and two positive siRNAs were used as the hit selection criteria. This further decreases the false-negative rate, although we can never completely exclude them.

### Reducing wtHTT

Lowering Mapk11 or Hipk3 reduces both mHtt and wtHtt in HD cells (Figure 2). This raises an important concern over the tolerance of losing wtHTT, which is essential for embryonic development^15,16,62^. Consistently, it has been reported that compound heterozygous variants in *HTT* are found in association with an autosomal recessive neurodevelopmental disorder^63^. Presence of a single-nucleotide polymorphism on the WT *HTT* allele that represses *HTT* transcription is associated with an earlier age of onset in HD patients in an extreme case-based cohort^17^. A very recent study also suggested that very low levels of wtHtt in mice during embryonic and postnatal development may lead to HD-like phenotypes later^64^. These studies suggest that loss of wtHTT to a certain extent may be harmful and may contribute to HD as well. On the other hand, lowering wtHTT levels up to 60%-70% has been shown to be safe in a primate model^65^ and ablation of wtHTT in adult mice at > 4 months of age does not lead to neurodegeneration or other defects in mouse models^66^, suggesting that lowering wtHTT in old adults is well tolerated. Consistent with this, HTT-lowering by RNAi or ASOs targeting both mHTT and wtHTT showed significant benefits in HD models^7,8,9,67,68^, confirming lowering both mHTT and wtHTT as a potential strategy for HD treatment. Encouragingly, the IONIS HTT-Rx clinical trial has been launched, using a non-allele specific ASO, HTTRx, for HD treatment (https://www.clinicaltrials.gov/ct2/show/NCT02519036). Another appealing strategy is to use allele-specific HTT ASOs to selectively lower mHTT^36,69,70,71^, although whether HD-relevant phenotypes *in vivo* could be rescued by the allele-specific strategy needs to be further addressed. Taken together, while lowering both mHTT and wtHTT to a certain extent may be beneficial, precautions need to be taken due to the potential harmful effects of reducing wtHTT to an intolerable extent.

In our study, lowering Mapk11 or Hipk3 reduces wtHtt by the extent of < 50% *in vivo* (Supplementary information, Figure S6B), and the reduction of wtHTT level appears within its tolerable range according to previous studies. Indeed, lowering Mapk11 by genetic silencing led to rescue rather than exacerbation of phenotypes in HD mice and cells (Figure 8).

We believe that mHtt lowering *per se* is likely to be the major mechanism that mediates the effect of Mapk11 on HD-relevant phenotypes, given the compelling evidence showing that reducing mHtt by many different approaches including directly knocking down Htt can rescue HD-relevant phenotypes^5,6,7,8,68^. This is because the potential diverse secondary effects from these approaches are unlikely to lead to rescuing effects through a common mechanism. Importantly, although lowering Mapk11 has no positive effects in WT mice in the behavioral assays, it rescues HD mice (Figure 8), suggesting that the effect is likely mediated through the lowering of mHtt. However, it is impossible to entirely exclude potential secondary effects that may have also been involved. Nevertheless, identification of Mapk11 as a potential druggable target to lower mHtt and associated toxicity warrants its further study for HD therapy.

### HD mouse models

Currently over a dozen HD mouse models have been established for various studies, including N-terminal HTT fragment models that include N171-82Q and R6 mice^72,73^; full-length transgenic models such as BACHD or YAC128^74,75^; or KI models such as Q140 and zQ175^34,76^. Each model has its own characteristics and advantages depending on the experimental approach to be taken. In this study, we chose to use a KI model, which expresses a human-mouse chimeric mHtt protein from the original locus of *Htt* at endogenous levels^34^ and this faithfully depicts HD genetics. Similar to human patients, the polyQ length in the KI mouse models correlates well with the degree and the age of onset of HD-relevant transcriptional dysregulation and behavioral deficits^77,78^, thus confirming KI mice as a reliable HD model. Among the commonly used KI models, the zQ175 model expresses the longest polyQ tract and has the strongest phenotypes^76^, but this model shows significant weight loss and the age of onset of behavioral deficits is around 4.5 months (20-30 years in human age), much earlier than the age of onset in most HD patients (40-50 years old). The KI model that we used in this study, *Hdh*Q140^34^, has relatively mild behavioral phenotypes which are only observable in homozygous mice at least under our experimental conditions (Figure 8). On the other hand, while all the other models mentioned above display weight changes, the *Hdh*Q140 model does^34^, thus minimizing potential artifacts from this aspect. In addition, the age of onset of behavioral deficits (∼7 months) in this model is analogous to that of HD patients. Particularly, our study has revealed that Mapk11 regulates endogenous *Htt* mRNA levels. Given that KI mice express mHtt from the endogenous mouse *Htt* promoter^34^, we reason that KI models are well suited for our study to examine the effects of Mapk11. Thus, we utilized *Hdh*Q140 for our first *in vivo* validation.

### MAPK and polyQ proteins

MAPK11 regulates HTT levels by regulating stability of its mRNA while HIPK3 regulates mHTT via autophagy. As a novel modulator of autophagy, HIPK3 may not only be able to regulate mHTT, but also other disease proteins degraded by autophagy. Interestingly, wtHTT could also be regulated by HIPK3 in the presence of mHTT (Figures 2 and 3), suggesting that autophagy may partially mediate wtHTT degradation as well. This may not be surprising given that wtHTT protein participates in the autophagy process^79,80^. While we have revealed a mechanistic sketch of MAPK11-mediated and HIPK3-mediated HTT regulation, many mechanistic details remain to be identified. We discovered that MAPK11 mainly regulates *HTT* mRNA stability (Figure 5), but the downstream mechanism of its action is still unclear. Since MAPK family members are known to regulate RNA binding proteins and miRNAs^81,82^, we speculate that MAPK11 regulates *HTT* mRNA via certain RNA binding protein(s) and/or miRNA(s), which will need further study to confirm. Similarly, the mechanistic details need to be determined of how HIPK3 regulates autophagy through DAXX.

MAPKs can have critical and systematic involvement in regulating mHTT/polyQ proteins. Another hit from our screen, MAP2K6, is known to be a stress sensor upstream of MAPK11. HIPK3 has been shown to be elevated by JNKs^26^, which are also members of the MAPK family that mediate the stress response downstream of polyQ expansion proteins^83^. In addition, other members of the MAPK family have been shown to elevate the levels of mutant ATXN1^14^, another disease-causing polyQ expansion protein. The homolog of MAP2K6 in *Caenorhabditis elegans*, sek-1, has been shown to increase the expression of the polyQ expansion protein, pqn41-c, which leads to linker cell death during development^84^. Collectively, the MAPKs appear to play vital roles in regulating polyQ proteins that can lead to cell death during both disease and development, and they may provide a critical link between stress and cell death by regulating the polyQ-expansion proteins.

## Materials and Methods

### Plasmid constructs

The human HTT-exon1 constructs (Q72 and Q25) were synthesized by Thermo Fisher Scientific and subcloned into the pcDNA/DEST40 vector (Thermo Fisher Scientific, #12274-015). The mouse full-length *Hipk3* cDNA was codon-optimized and synthesized by Thermo Fisher Scientific and subcloned into the pTT5SH8Q2 vector (NRC Biotechnology) with N-terminal GFP tag. The human or rat *Hipk3* cDNA failed to express efficiently in *STHdh* cells. The Q72-GFP construct (expressing Methionine-Q72-GFP) was cloned by PCR amplification from the HTT-exon1 constructs, and ligation to the pcDNA-GFP plasmid. The QuikChange Site-Directed Mutagenesis Kit (Agilent Technologies) was used to generate the point mutation constructs: kinase-dead mouse Hipk3 (K226M and D322N), DN-MAPK11 (TGY (180-182) to AGF). The MAPK11 (#20355) and GFP-LC3B (#24987) construct was obtained from Addgene.

### Cell culture and cell line generation

*STHdh* (CH00097 – Q7/Q7 and CH00096 – Q111/Q7) and human fibroblasts (GM03868 – Q45; GM21757 – Q68) were obtained from Coriell Cell Repositories. The Q47 fibroblasts were obtained from a Mongolian HD patient, and the study was approved by the ethic community of IBS at Fudan University (No.28). Verbal and written consent was obtained from patients. *STHdh* cells were cultured in DMEM (Thermo Fisher Scientific, #11965) with 10% FBS (Thermo Fisher Scientific, #10082). Immortalized human patient fibroblasts were generated by SV40 large T-antigen and cultured in DMEM with 15% FBS. Primary fibroblasts were cultured under the same condition. Human ESC-derived neurons stably expressing HTT-exon1 fragments were generated and authenticated as previously reported^13^, and the generation and authentication of HD iPSC-derived neurons were described previously^29^. All the mammalian cell lines were maintained at 37 °C incubator with 5% CO_2_, except *STHdh* cells, which were maintained at 33 °C with 5% CO_2_. The cells were tested for mycoplasma contamination every month. Primary mouse neurons from striata acutely dissected from P0 pups and tissue was digested with Trypsin (Sigma, #T1005, 2 mg/ml) and DNaseI (Roche, #10104159001, 1 mg/ml) in DMEM for 30 min with occasional mixing. The digestion was then stopped by 10% serum, and the tissue was then triturated and plated in the Neurobasal A medium (Thermo Fisher Scientific, #21103049) supplemented with 1× B27 (Thermo Fisher Scientific, #17504044), 1× N2 (Thermo Fisher Scientific, #17502048), 0.5× Antibiotic-Antimycotic (Thermo Fisher Scientific, #15240062) and 1× Glutamax (Thermo Fisher Scientific, #35050061)

### Neuronal loss and apoptosis measurements

The secondary screen for neuronal loss of hESC-derived HD neurons was performed similarly to a previously study^13^. Basically, the neurons were moved from protective culture condition to standard culture condition and displayed mHTT-dependent neuronal shrinkage and death, which could be quantitated by confluence measurement using incuCyte (Essen Biosciences)^13^. For measurements of caspase-3 activity of the iPSC-derived neurons, the NucView 488 caspase-3 dye (Biotium, #30029) was used for the caspase 3 activity detection as an indicator of apoptosis. The images of the caspase-3 dye treated cells were taken by IncuCyte automatically. The phase-contrast images were then taken by the IncuCyte equipment (Essen BioScience) and analyzed for confluence by the IncuCyte software.

### Protein extraction and western blots

Cell pellets were collected and lysed on ice for 30 min in 1× PBS+1% Triton X-100+1× Complete protease inhibitor (Roche), sonicated for 10 s, and centrifuged at > 20 000× *g* at 4 ^o^C for 10 min. The supernatants were then loaded and transferred onto nitrocellulose membranes for western blots. For mouse brain tissues, the striata and cortices were dissected on ice and ground with a tissue grinder for 5 min at 60 Hz and lysed on ice for 60 min in brain lysis buffer (50 mM Tris, 250 mM NaCl, 5 mM EDTA, 1% Triton X-100 pH=7.4) + 1× Complete protease inhibitor (Roche). The samples were then spun at > 20 000× *g* at 4 ^o^C for 15 min. The suspensions were then collected for western blots. The anti-HTT antibodies 2B7^18^, ab1^85^ and MW1^86^ have been described previously. The antibody S830 for immunostaining of Htt aggregates is a kind gift from Dr Gillian Bates. Commercially purchased antibodies include anti-HTT antibodies 2166 (Millipore, #MAB2166, RRID:AB_2123255), 3B5H10 (Sigma, #P1874), anti-β-tubulin (Abcam, #ab6046, RRID:AB_2210370), anti-Ataxin3 (Millipore, #MAB5360), anti-Mapk11 (Thermofisher Scientific, #33-8700), anti-Tuj1 (Covance, #MMS-435P, RRID:AB_2313773), anti-Darpp32 (Abcam, #ab40801, RRID:AB_731843), anti-P62 (Abcam, #ab56416, RRID:AB_945626), anti-DAXX (Proteintech, #20489-1-AP, RRID:AB_10693620), anti-LC3B (Proteintech, #18725-1-AP), anti-HIPK3 (Santa cruz, #sc-66920, RRID:AB_2117734) ), anti-β-actin (Abcam, #ab6276), anti-Calnexin (Enzo, #ADI-SPA-860-F), anti-Gapdh (Santa cruz, #sc-25778), anti-α-tubulin (Proteintech, #11224-1-AP), anti-MAP2 (Santa cruz, #sc-20172), anti-phospho-ATF2 (Cell Signaling Technology, #9225, RRID:AB_2060933), anti-ATF2 (Cell Signaling Technology, #9226, RRID:AB_2060930), anti-Phospho-p38 (Cell Signaling Technology, #4511, RRID:AB_2139682), anti-Beclin1 (Thermofisher, #PA1-46464) and anti-LC3 antibody that preferentially detects LC3II (Thermofisher, #70012)^52^. The specificity of all antibodies has been validated by previous reports or our knockdown or knockout experiments.

### Homogeneous time-resolved fluorescence assay

The HTRF assays were performed similarly to those previously described^30^. Basically, the cells were lysed in the lysis buffer (1× PBS with 0.4% Triton X-100 and 1× protease inhibitor final) and the target detected with indicated antibody pairs. The principles are explained in Supplementary information, Figure S1. Note that the 2B7/MW1 antibody pair is needed to test mHTT in human cells, while this antibody pair does not work properly in mouse *STHdh* cells. Another antibody pair 2B7/2166 was used to measure Htt levels (wtHtt and mHtt) in mouse *STHdh* cells^28^. This antibody pair could also be used to detect wtHTT proteins in the WT cells or HTT proteins including both mHTT and wtHTT in the HD cells^21^. For all samples, the total protein concentration (by BCA, Pierce, #23225) and/or the total DNA content (by Picogreen, #P7589) were measured to correct the loadings. Different protein concentrations or cell numbers per well were tested to ensure that the signals were in the linear range. Background corrections were performed by subtracting the background signals from blank samples.

### cDNA and siRNA transfection

The siRNAs were reversely transfected into the *STHdh* cells with lipofectamine 2000 (Life Technologies, #11668) and into the iPSC-derived neurons with lipofectamine RNAiMAX (Life Technologies, #13778) whereas cDNAs were transfected with lipofectamine 3000 (Life Technologies, # L3000). All transfections were performed according to the manufacturer's protocol. Cells were collected 3 days after siRNA transfection or 2 days after cDNA transfection for western blot, HTRF or Immunofluorescence. All siRNAs have been validated by qPCR and/or western blots for target knockdown. For the siRNA target sequences, see Supplementary information, Data S1.

### Mouse models

The generation and characterization of the *Hdh*140Q KI mice have been previously described^34^. Mice were provided by Dr. Marian DiFiglia at Massachusetts's General Hospital, USA. The mice were then bred at the mouse facility at Fudan University. *Mapk11* and *Hipk3* knockouts were generated by Cyagen Biosciences Inc. using TALEN technology. The knockout mice were produced by microinjecting TALEN mRNAs into fertilized eggs from the C57BL/6 strain. The knockout alleles were validated by sequencing for an early frameshift of the target genes as well as western blots (Supplementary information, Figure S5). The mice were back-crossed to the C57BL/6 WT background for five generations before crossing to the HD mice (with the same genetic background) or performing other experiments. Mice were group-housed (up to five adult mice per cage) in individually vented cages with a 12 h light/dark cycle. The mouse experiments were carried out following the general guidelines published by the Association for Assessment and Accreditation of Laboratory Animal Care. The Animal Care and Use Committee of the School of Medicine at Fudan University approved the protocol used in animal experiments (Approval #20140904). For protein extraction from the mouse brain, the brains were collected and the striata and cortices were acutely dissected.

### RT-qPCR

mRNA levels were determined by qPCR. RNA from siRNA-transfected or compound-treated cells was extracted using RNAprep Pure Cell/Bacteria Kit (Tiangen, #DP430). DNase I was added to break down the genomic DNA. Random-primed cDNA was obtained by reverse transcription using the FastQuant RT Kit (Tiangen, #KR106). qPCR was then performed using SYBR Green Realtime PCR Master Mix (Toyobo, #QPK-201). All the primers were tested with standard curve, amplification efficiency was between 95% and 105%, and the *R*^2^ for linear relationship is > 0.999. No reverse-transcriptase controls were used to ensure the specificity of the signals.

mRNA stability measurements were similar as previously described^87^. The cultured cells were treated with transcriptional inhibitor actinomycin D (5 μg/ml, Sigma, #A9315) and the cells were collected at indicated time points after treatment for measurement of HTT mRNA levels by RT-qPCR.

For all the qPCR primer sequences see Supplementary information, Data S1.

### Nonradioactive pulse-chase experiments

The Click-iT AHA (ℒ-azidohomoalaine) kit (Thermo Fisher Scientific, #C10102) was used and the assay was similar to that previously described^30,88^. Briefly, cells were starved with methionine-free media for 1 h prior to adding Click-iT AHA for 12 h (pulse, 50 μM). The cells were then incubated in normal serum-containing media for the indicated periods before harvesting (chase) for analysis. The Click-iT AHA labeled proteins were then conjugated with biotin by treatment with biotin-DIBO Alkyne (Thermo Fisher Scientific, #C10412). The biotin conjugated proteins were then pulled-down using streptavidin beads (Pierce, #20349), and the HTT level is determined by HTRF^88^.

### *HTT* promoter transcription assay

The assay was performed similarly to that previously described^48^. Basically, cells were transfected with the reporter plasmid expressing firefly luciferase driven by the human *HTT* promoter (pHTT-A) mixed with control plasmid expressing renilla luciferase (pRL-TK) using lipofectamine 3000. The pHTT-A and pRL-TK plasmids were co-transfected at 10:1 ratio. After 48 h of transfection, firefly and renilla luciferase activities were measured using the dual-glo luciferase reporter assay system (Promega, #E2920). The firefly luciferase activity was normalized to the renilla luciferase activity to reflect the promoter activity.

### Compound treatment

For compound treatment, the cells were plated at the same density as the one for siRNA transfection, and the compounds were diluted in OPTI-MEM in 10× concentrations and added 1-2 days later. The cells were then collected after 1-2 days for further analysis. Compounds were obtained as follows: bafilomycin A (Sigma, #B1793, 100 nM), epoxomicin (Cayman Chemical, #BU4061T, 100 nM), MG132 (Sigma, #M7449, 1 μM), ammonium chloride (Sigma, #A9434, 10 mM), SB202190 (Sigma, #152121-30-7, at indicated concentrations), DPI (Sigma, #D2926, 100 nM, at indicated concentrations) and AST487 (Chemexpress, #HY-15002, at indicated concentrations).

### Immunofluorescence and immunohistochemistry

For immunofluorescence, cultured cells were fixed in 4% paraformaldehyde (PFA) after washing with 1× PBS for three times, and then permeablizing in 0.5% TritonX-100 for 10 min. The cells were then blocked in 4% BSA + 0.1% Triton X-100 in 1× PBS for 30 min and incubated overnight at 4 °C with primary antibodies, and then washed three times with blocking buffer and incubated with secondary antibody at room temperature for 1 h. Coverslips were then washed three times, stained with 0.5 mg/ml DAPI (Beyotime Biotechnology, #C1002) for 5 min at room temperature, and then mounted in vectashield mounting medium (Vector, #H-1002).

For mouse brain slices, mice were killed and then perfused with PBS and 4% paraformaldehyde. The brains were then dissected out, and fixed with 4% PFA for 48 h at 4 °C. The brains were then incubated in 30% sucrose for 48 h at 4 °C and sectioned at −20 °C into 35 μm thick slices. The immunofluorescence experiments were then performed as described above for cells except substituting 3% donkey serum for blocking.

Sample brain slices from human patients were obtained from Addenbrookes Hospital (Cambridge, UK) with an appropriate ethical approval from Brain UK (14/007). The slices were deparaffinized with dimethylbenzene, rehydrated with reverse gradient ethanol and then boiled in sodium citrate buffer (pH = 6.0) for antigen retrieval. Endogenous peroxidase was then quenched with 3% H_2_O_2_. Incubation with primary antibodies (Hipk3: GeneTex, #GTX108369 and Mapk11: Thermofisher Scientific #33-8700) was at 4 °C overnight after blocking with 4% goat serum. Detection was with peroxidase streptavidin-biotinylated complex and diaminobenzidine (DAB)/H_2_O_2_ (Boster, #SA1050). Experiments without primary antibodies and knockdown experiments in cells were utilized to confirm the specificity of the antibodies. Images were taken using Zeiss LSM confocal microscopes.

### Mouse behavioral experiments

All the behavioral experiments were performed during the light phase and the experimenters were blind to the genotype of each mouse. Both males and females were used. All the mice were kept in the behavioral test room in dim red light for 1 h before starting the experiments. For activity tests, mice were placed in a pen holder with a mashed surface (98 mm height × 91 mm diameter) for 5 min. The activity number indicates the total number of active behaviors including rearing (lifting two paws, most frequent) and climbing (lifting three or more paws onto the wall, rare). For open-field tests, mice were placed in a 30 × 30 × 40 cm white Plexiglas chamber (equipment from Med-Associates) in a behavior room, and locomotion was captured by camera on top of the chamber and recorded for 15 min. The traveling trace and distance were then analyzed using the Med-Associates Activity Monitor program. For gait analysis, CatWalk XT was used as a complete system for quantitative assessment of the gait of mice. Mice were placed in an enclosed walkway on a glass plate and allowed to traverse from one side of the walkway to the other. Their motion and footprints were captured by a high speed video camera positioned underneath the walkway. The equipment was set so that failure to move to the other side within 9 sec was considered as a failure, not suitable for quantification. Three successful trials were performed for each mouse and the data were analyzed with the CatWalk software to obtain the different parameters. The principle component analysis was based on the average of three trials for each mouse. Some of the animals were tested by all three behavioral assays whenever time and equipment availability allowed.

### Statistical analysis

Statistical comparisons between two groups were conducted by the two-tailed unpaired Student's *t*-tests. Statistical comparisons among multiple groups were conducted by ANOVA tests. Significance was established at *P*< 0.05. In all graphs, error bars indicate SEM, and the biological replicate numbers are indicated on top of each bar and/or as the *n* numbers in the legends. The statistical powers for all analyses were calculated and confirmed to be > 80%. The key experiments including detecting Htt level changes upon *Mapk11/Hipk3* knockdown or knockout and the analysis of aggregates or LC3 puncta were confirmed by blind tests in which different investigators prepared/labeled the samples and the analyses were carried out without information exchange before obtaining the results. The mouse behavioral experiments were all performed in completely blind manner. Data were excluded when there were clear indications of artifact or experimental failures, such as contamination, transfection/infection failure, etc.

## Author Contributions

BL initiated the project and designed the experiments; MY, Y F, YL, PW carried out the experiments in cell cultures and mouse tissues; YL performed immunohistochemistry in human putamen slices; BL, YY, LM and YF performed the experiments in human stem cell-derived neurons; MY and HS carried out the mouse behavioral experiments; XW performed molecular cloning; SL provided essential reagents and help with autophagy-relevant experiments.

## Competing Financial Interests

The authors declare no competing financial interest.

## Supplementary Information

Supplementary information, Figure S1 The HTRF assay for soluble Htt measurements

Supplementary information, Figure S2 Validation of siRNAs

Supplementary information, Figure S3 Expression of HIPK3 and MAPK11 in human and mouse brains

Supplementary information, Figure S4 Mapk11 and Hipk3 knockout mice

Supplementary information, Figure S5 Lowering Mapk11 or Hipk3 does not reduce control protein levels.

Supplementary information, Figure S6 Mapk11/Hipk3's effect on mHtt and wtHtt and controls for mHtt-dependence.

Supplementary information, Figure S7 Hipk3 regulates HTT levels via autophagy.

Supplementary information, Figure S8 Rationale for using homozygous Q140 mice for phenotypic validation of Mapk11

Supplementary information, Table S1 Component Score Coefficient Matrix

Supplementary information, Table S2 Supplementary to Figure 8: gait analysis using single parameters

Supplementary information Data S1

Supplementary information, Movie S1 Representative gait recordings of *Hdh*^Q7/Q7^

Supplementary information, Movie S2 Representative gait recordings of *Hdh*^Q140/Q140^
