# Supplementary material for: Suppression of MAPK11 or HIPK3 reduces mutant Huntingtin levels in Huntington's disease models
Source: Cell Res. 2017 Oct 13;27(12):1441–65. doi: 10.1038/cr.2017.113 (PMC5717400; doi:10.1038/cr.2017.113)
Supplement: Supplementary information, Figure S1 — The HTRF assay for soluble Htt measurements [file cr2017113x1.pdf]

Figure S1

A

Principles for HTRF

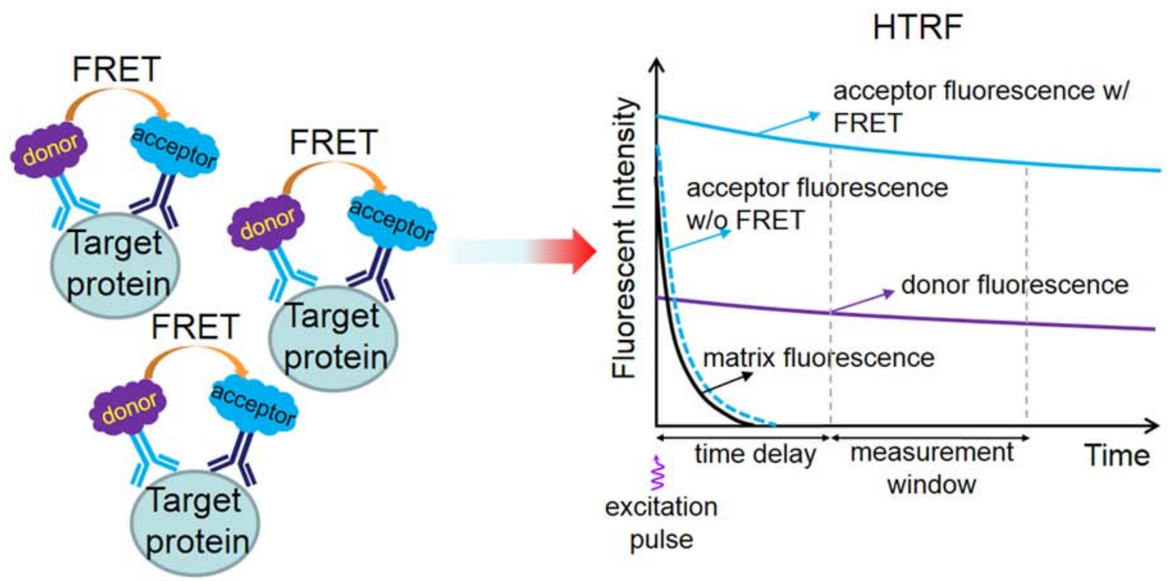

B

Antibody pairs utilized in this study

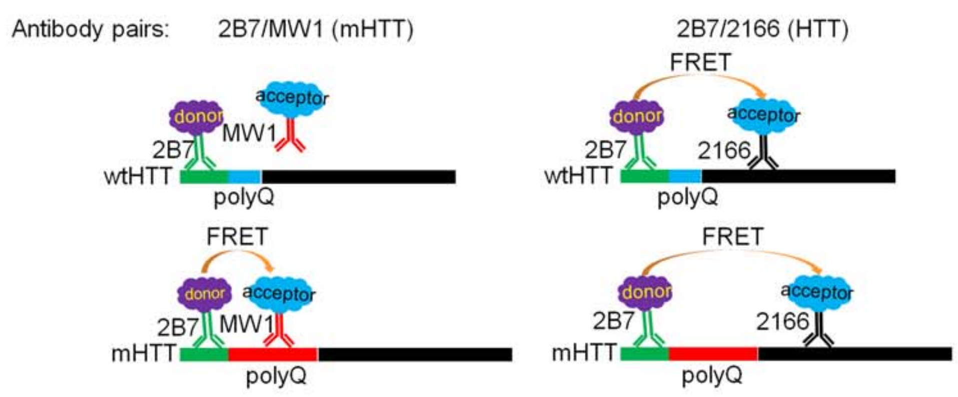

Figure S1-supplementary to Figure 1: The HTRF assay for soluble Htt measurements

(**A**) The HTRF technology is based on Fluorescence Resonance Energy Transfer (FRET), which is the transfer of energy between two fluorophores in close proximity (5~9 nm), referred to as a donor and an acceptor (left). Excitation of the donor by an energy source triggers an energy transfer towards the acceptor, which in turn emits a specific fluorescence. Traditional FRET signals are influenced by background fluorescence from sample components, which is extremely transient and thus can be eliminated in HTRF: it uses a rare earth complex as the donor, which gives long-lived fluorescence signals peak at 615 nm upon excitation by an energy pulse. As a result, HTRF acceptors emit long-lived fluorescence peak at 665 nm only when engaged in a FRET process. Thus, a time delay of approximately 50 to 150 μs between the system excitation and fluorescence measurement could be introduced to eliminate non-FRET short-lived emissions (right). By using the acceptor conjugated and the donor conjugated antibodies targeting the same protein, the HTRF signals are generated only when the two antibodies bind with the same protein molecule. Therefore the signals are in proportion to the target protein concentration and could be used to quantify its level.

(**B**) The 2B7/MW1 antibody pair detects mHTT only, because the MW1 antibody has much higher apparent affinity to long polyQ. The 2B7/2166 antibody pair detects the total signal from both wtHTT and mHTT, because the 2166 antibody interacts with both. These HTRF antibody pairs have been validated for measurements of HTT/Htt protein levels in previous studies (PMID19664996, 22996692, 22365609,25300329) and they have been utilized in many other published studies. For mouse striatal cells (STHdh), 2B7/MW1 can not be used due to non-specific signals, and only the 2B7/2166 antibody pair could be used (PMID 25300329).
