# Supplementary material for: Suppression of MAPK11 or HIPK3 reduces mutant Huntingtin levels in Huntington's disease models
Source: Cell Res. 2017 Oct 13;27(12):1441–65. doi: 10.1038/cr.2017.113 (PMC5717400; doi:10.1038/cr.2017.113)
Supplement: Supplementary information, Figure S2 — Validation of siRNAs [file cr2017113x2.pdf]

Figure S2

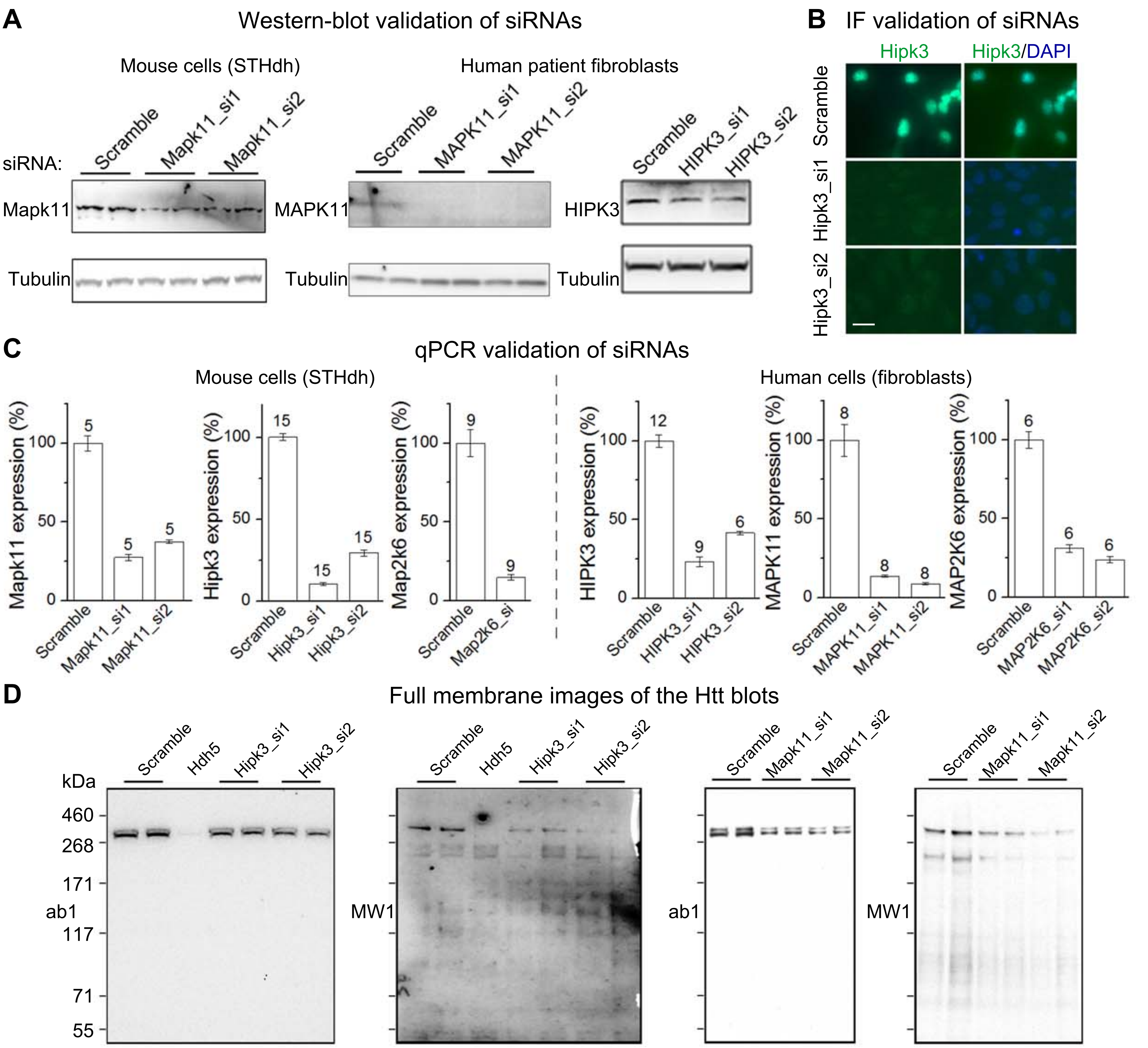

Figure S2 - Supplementary to Figure 2: Validation of siRNAs

(A) Representative western-blots showing the knock-down of target by Mapk11/MAPK11 siRNAs in both mouse (STHdh) and human (fibroblasts) cells, and HIPK3 siRNAs in human cells (fibroblasts). Due to many non-specific bands around 80 kD and low antibody affinity, we failed to detect mouse endogenous Hipk3 protein-specific western-blot signals in STHdh cells.

(B) Immunofluorescence (IF) experiments confirming the expression and knock-down of Hipk3 proteins in the STHdh cells. *Scale* bar: 20 μm. The anti-Hipk3 antibody from GeneTex (#GTX108369) was used as the primary antibody, and the same antibody was used for human putamen slices (Figure S3E). This antibody fails to give signals in western-blot experiments.

(C) qPCR results showing the knock-down of target by the kinase siRNAs used in this study. Different siRNAs were used for human and mouse targets and validated in mouse (*left* three panels) and human cells (*right* three panels), respectively. Expression is normalized to the Scramble siRNA transfected control sample. Data are plotted as mean and s.e.m.

(D) Full-membrane blots of the Htt western-blots in Fig. 2A showing that the reduction of full length Htt is not due to increase of cleavage products.
