# Supplementary material for: Suppression of MAPK11 or HIPK3 reduces mutant Huntingtin levels in Huntington's disease models
Source: Cell Res. 2017 Oct 13;27(12):1441–65. doi: 10.1038/cr.2017.113 (PMC5717400; doi:10.1038/cr.2017.113)
Supplement: Supplementary information, Figure S3 — Expression of HIPK3 and MAPK11 in human and mouse brains [file cr2017113x3.pdf]

Figure S3

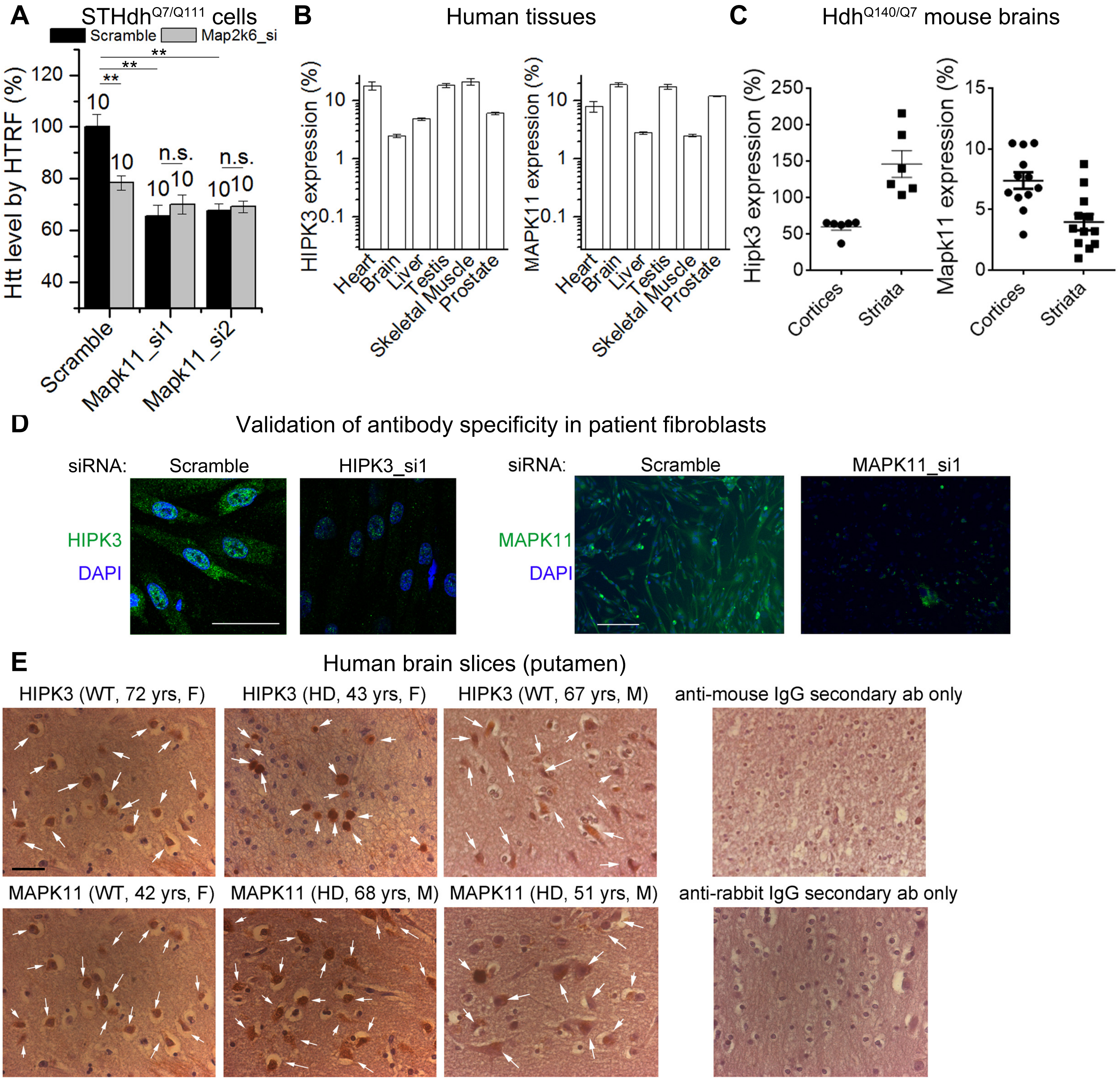
A
STHdh^Q7/Q111 cells
Scramble
Map2k6_si
Htt level by HTRF (%)
n.s.
Scramble
Mapk11_si1
Mapk11_si2
B
Human tissues
HIPK3 expression (%)
MAPK11 expression (%)
Heart
Brain
Liver
Testis
Skeletal Muscle
Prostate
C
Hdh^Q140/Q7 mouse brains
Hipk3 expression (%)
Mapk11 expression (%)
Cortices
Striata
D
Validation of antibody specificity in patient fibroblasts
siRNA:
Scramble
HIPK3_si1
HIPK3
DAPI
siRNA:
Scramble
MAPK11_si1
MAPK11
DAPI
E
Human brain slices (putamen)
HIPK3 (WT, 72 yrs, F)
HIPK3 (HD, 43 yrs, F)
HIPK3 (WT, 67 yrs, M)
anti-mouse IgG secondary ab only
MAPK11 (WT, 42 yrs, F)
MAPK11 (HD, 68 yrs, M)
MAPK11 (HD, 51 yrs, M)
anti-rabbit IgG secondary ab only

Figure S3-Supplement to Figure 2: Expression of HIPK3 and MAPK11 in human and mouse brains
(A) Measurement of Htt levels by HTRF using the 2B7/2166 antibody pair in the STHdh$^{Q7/Q111}$ cells transfected with the indicated siRNAs. Transfection of Mapk11 siRNAs or Map2k6 siRNAs reduced Htt levels. In addition, transfection of Mapk11 siRNA diminished the effect of Map2k6 siRNA on Htt levels, suggesting that Mapk11 is downstream of Map2k6. The statistical analysis was performed by two-tailed unpaired t tests.
(B) qPCR results showing the expression of HIPK3 and MAPK11 mRNA normalized to HPRT in various human tissues (n = 3). The human cDNAs from each tissue were purchased from Clonetech (#636742).
(C) qPCR results showing the expression of Hipk3 and Mapk11 mRNA (normalized to HPRT) in HD mouse (HdhQ7/Q140 knock-in) cortices and striata. The ages of mice are the same as in Fig. 2C-D: 10 months old for the Hipk3 expression detection and 4~6.5 months for the Mapk11 expression detection.
(D) Antibody specificity was validated by immunofluorescence of human patient fibroblasts transfected with HIPK3 or MAPK11 siRNAs versus the scrambled control. The signals were reduced by knock-down, confirming antibody specificity. *Scale* bar: 50 μm.
(E) Immunohistochemistry staining of MAPK11 and HIPK3 in putamen slices from HD versus control (WT) postmortem patients with indicated ages. The brown color indicates expression of the target protein. The dark blue color indicates nuclei. The antibody specificity has been validated by control slices with secondary antibody alone (*right* panels). The information of each patient is indicated on top of each image. The positive signals are pointed by white arrowheads. WT: patients without Huntington’s disease; HD: patients with Huntington’s disease (at Grade III); F: female. M: male; *Scale* bar: 50 μm. Over 20 slices from 6 people have been stained and imaged for each protein, and all slices tested showed expression.
