# Supplementary material for: Suppression of MAPK11 or HIPK3 reduces mutant Huntingtin levels in Huntington's disease models
Source: Cell Res. 2017 Oct 13;27(12):1441–65. doi: 10.1038/cr.2017.113 (PMC5717400; doi:10.1038/cr.2017.113)
Supplement: Supplementary information, Figure S4 — Mapk11 and Hipk3 knockout mice [file cr2017113x4.pdf]

Figure S4

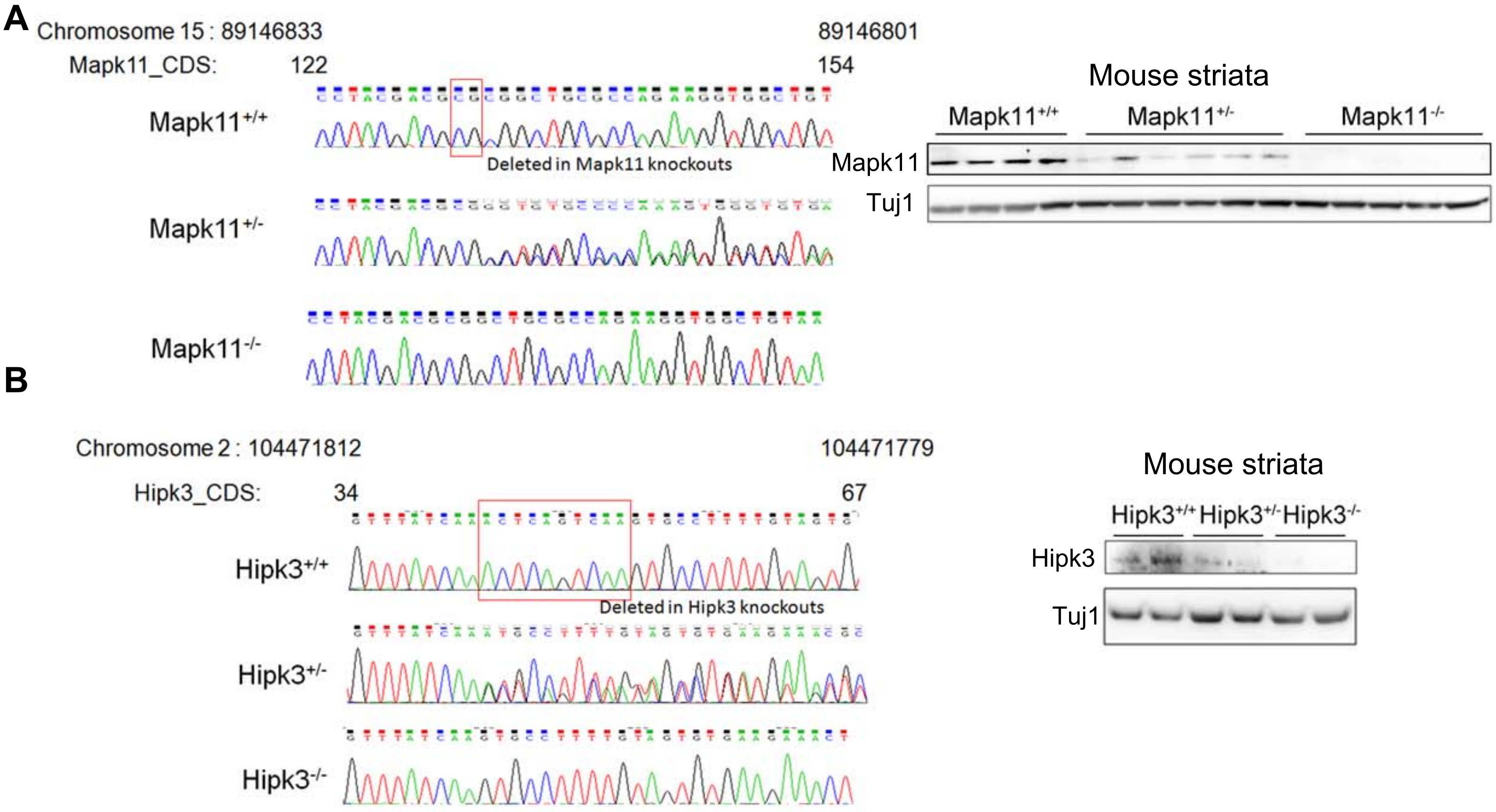

Figure S4-Supplementary to Figure 2: Mapk11 and Hipk3 knockout mice

(A) *Left***:** Representative sequencing results for Mapk11$^{+/+}$, Mapk11$^{+/-}$ and Mapk11$^{-/-}$ mice. The red box indicates the sequence that is missing in the knockout animal. *Right*: Representative western-blots from mouse striata of the indicated genotypes showing the knockout of Mapk11 protein

(B) Similar as (A), but for validation of Hipk3 knockouts. Note that both these genes are in the reverse orientation in the genome. For western-blots, the loading per lane is 20 µg for Mapk11 and 50 µg for Hipk3.
