# Supplementary material for: Suppression of MAPK11 or HIPK3 reduces mutant Huntingtin levels in Huntington's disease models
Source: Cell Res. 2017 Oct 13;27(12):1441–65. doi: 10.1038/cr.2017.113 (PMC5717400; doi:10.1038/cr.2017.113)
Supplement: Supplementary information, Figure S5 — Lowering Mapk11 or Hipk3 does not reduce control protein levels. [file cr2017113x5.pdf]

Figure S5

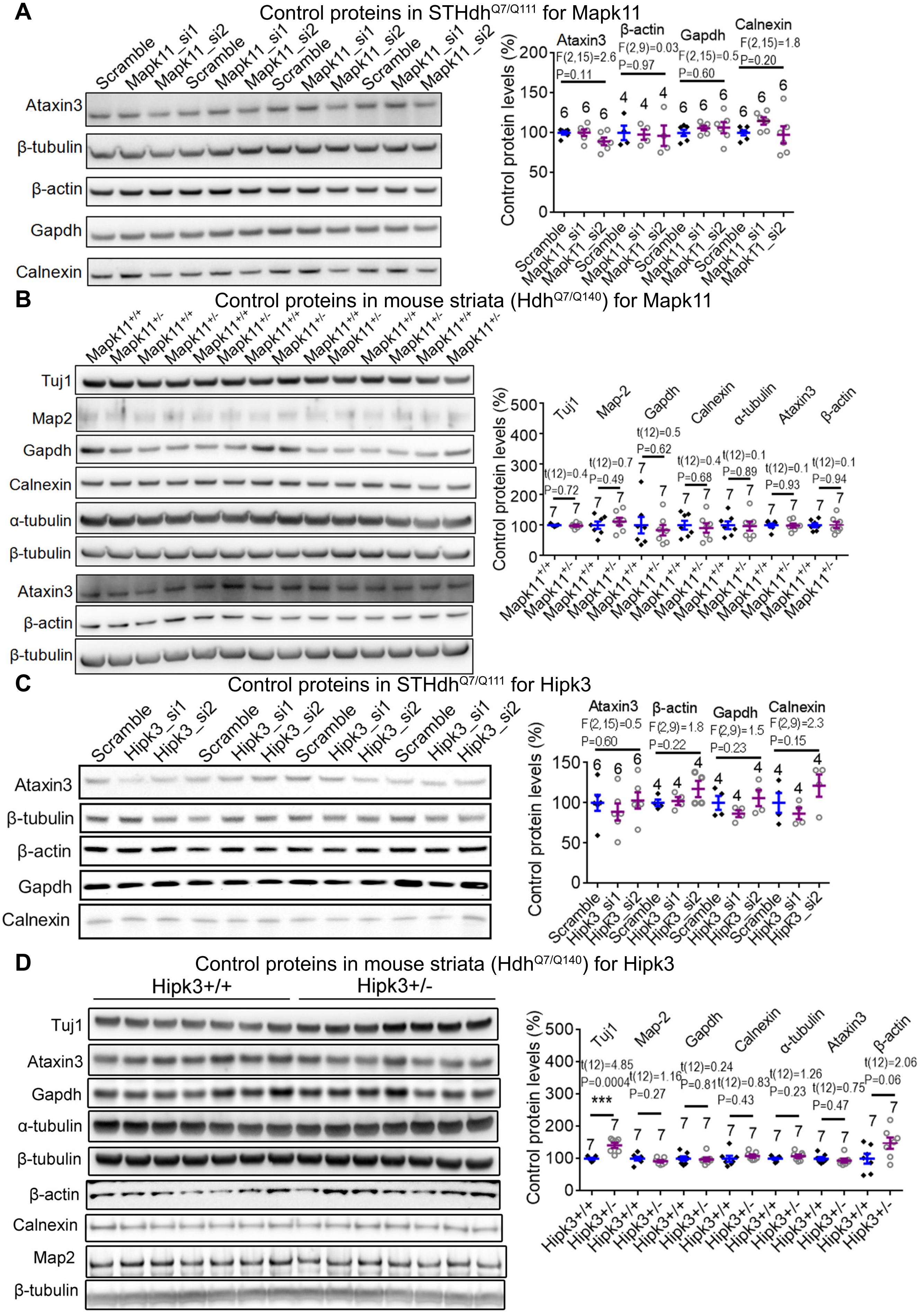

A
Control proteins in STHdh Q7/Q111 for Mapk11
Scramble
Mapk11_si1
Mapk11_si2
Ataxin3
β-tubulin
β-actin
Gapdh
Calnexin
Control protein levels (%)
Ataxin3
F(2,15)=2.6
P=0.11
β-actin
F(2,9)=0.03
P=0.97
Gapdh
F(2,15)=0.5
P=0.60
Calnexin
F(2,15)=1.8
P=0.20
B
Control proteins in mouse striata (Hdh Q7/Q140) for Mapk11
Mapk11+/+
Mapk11+/-
Tuj1
Map2
Gapdh
Calnexin
α-tubulin
β-tubulin
Ataxin3
β-actin
Control protein levels (%)
Map-2
t(12)=0.4
P=0.72
t(12)=0.7
P=0.49
t(12)=0.5
P=0.62
t(12)=0.4
P=0.68
t(12)=0.1
P=0.89
t(12)=0.1
P=0.93
t(12)=0.1
P=0.94
C
Control proteins in STHdh Q7/Q111 for Hipk3
Scramble
Hipk3_si1
Hipk3_si2
Ataxin3
β-tubulin
β-actin
Gapdh
Calnexin
Control protein levels (%)
F(2,15)=0.5
P=0.60
F(2,9)=1.8
P=0.22
F(2,9)=1.5
P=0.23
F(2,9)=2.3
P=0.15
D
Control proteins in mouse striata (Hdh Q7/Q140) for Hipk3
Hipk3+/+
Hipk3+/-
Tuj1
Ataxin3
Gapdh
α-tubulin
β-tubulin
β-actin
Calnexin
Map2
Control protein levels (%)
Map-2
t(12)=4.85
P=0.0004
***
t(12)=1.16
P=0.27
t(12)=0.24
P=0.81
t(12)=0.83
P=0.43
t(12)=1.26
P=0.23
t(12)=0.75
P=0.47
t(12)=2.06
P=0.06

Figure S5: Lowering Mapk11 or Hipk3 does not reduce control protein levels.
(A) Western-blots and quantification (normalized to the β-tubulin signals) of control proteins in $STHdh^{Q7/Q111}$ cells transfected with the indicated siRNAs (Mapk11 siRNAs versus the control). The statistical analysis was performed by one-way ANOVA. No significance ($P<0.05$) was detected, and thus no post hoc analysis could to be performed. The n, F and p values are indicated on top of the plots. Colored lines indicate mean and SEM.
(B) Western-blots and quantification (normalized to the β-tubulin signals) of control proteins in HD mouse ($Hdh^{Q7/Q140}$) striata with the indicated Mapk11 genotypes. The statistical analysis was performed by two-tailed unpaired t tests. The mice were the same as Figure 2D (6.5m). The n, t and p values are indicated on top of the plots. Colored lines indicate mean and SEM.
(C) Similar as (A), but transfected with Hipk3 siRNAs versus the control.
(D) Similar as (B), with the indicated Hipk3 genotypes. The mice were the same as Figure 2C.
