# Supplementary material for: Suppression of MAPK11 or HIPK3 reduces mutant Huntingtin levels in Huntington's disease models
Source: Cell Res. 2017 Oct 13;27(12):1441–65. doi: 10.1038/cr.2017.113 (PMC5717400; doi:10.1038/cr.2017.113)
Supplement: Supplementary information, Figure S6 — Mapk11/Hipk3's effect on mHtt and wtHtt and controls for mHtt-dependence. [file cr2017113x6.pdf]

Figure S6

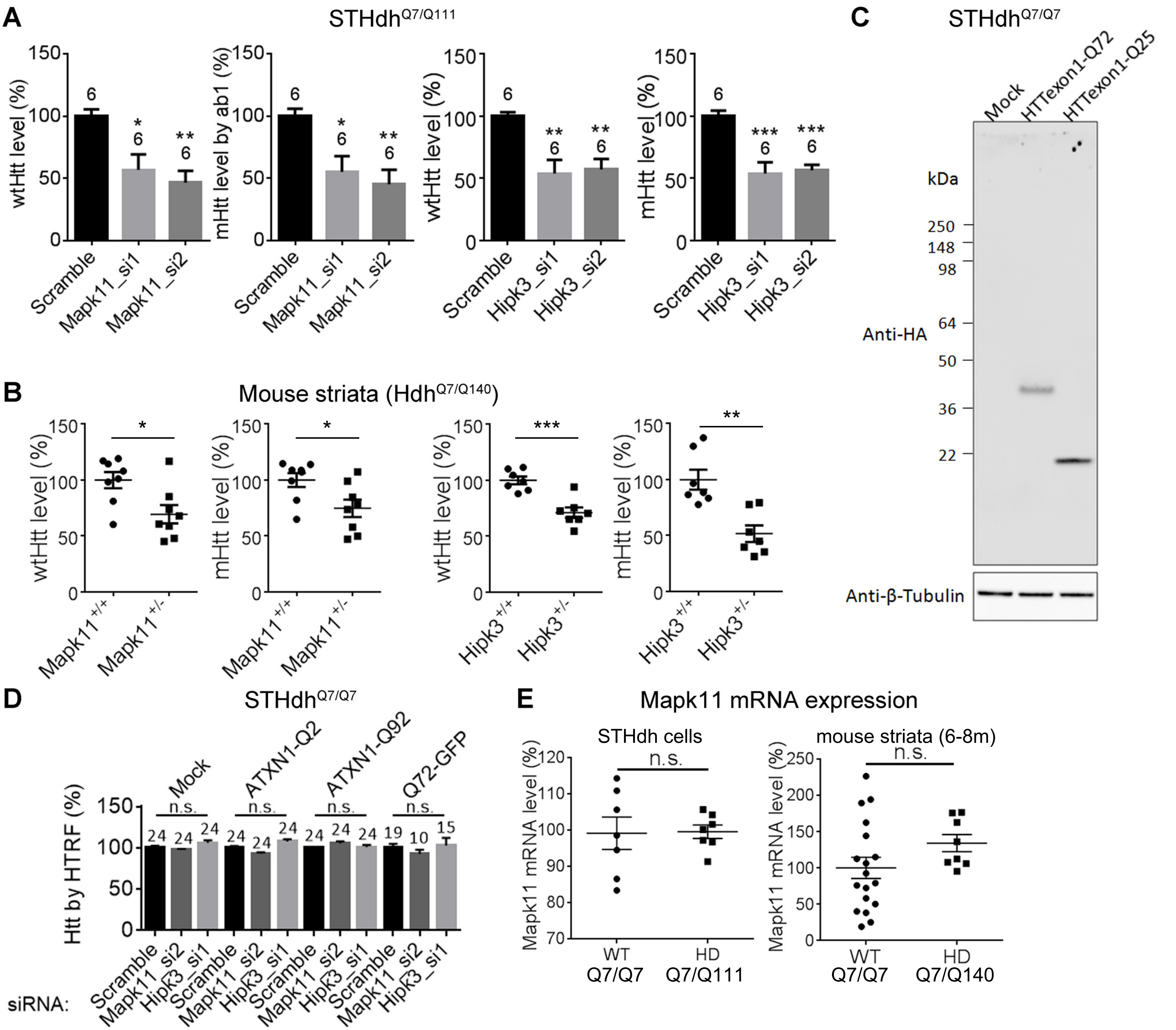

Figure S6-Supplementary to Figure 3: Mapk11/Hipk3's effect on mHtt and wtHtt and controls for mHtt-dependence.

(A) Quantifications of mHtt or wtHtt in STHdh$^{Q7/Q111}$ cells with indicated siRNA transfections. The quantifications are based on the western-blots presented in Figure 2A. The replicate number is slightly smaller than the quantifications in FIgure 2A, because the wtHtt and mHtt bands were very close and thus could not be quantified separately in two of the blots.Statistical analysis performed by one-way ANOVA and Dunnett's post hoc tests: *: P<0.05: **: P<0.01.

(B) Quantifications of mHtt or wtHtt in HD mouse striata of the indicated genotypes. Statistical analysis performed by unpaired two-tailed t tests: *: P<0.05,**: P<0.01.,***: P<0.001.

(C) Representative western-blots showing the expression of HTT-exon1 fragments.

(D) Htt levels measured by the 2B7/2166 HTRF in the STHdh$^{Q7/Q7}$ cells co-transfected with the indicated cDNA and the siRNAs indicated in the X-axis. No significant changes were observed, indicating that long polyQ alone (Q72) or other long polyQ proteins (Atxn1-Q92) is not sufficient to driven the MAPK11 or HIPK3 mediated Htt regulation. Statistical analysis was performed by one-way ANOVA tests.

(E) qPCR measurements of Mapk11 mRNA levels in HD versus WT cells or striata, and no signficant differences were detected. The statistical analysis was performed by two-tailed unpaired t tests.
