# Supplementary material for: Suppression of MAPK11 or HIPK3 reduces mutant Huntingtin levels in Huntington's disease models
Source: Cell Res. 2017 Oct 13;27(12):1441–65. doi: 10.1038/cr.2017.113 (PMC5717400; doi:10.1038/cr.2017.113)
Supplement: Supplementary information, Figure S7 — Hipk3 regulates HTT levels via autophagy. [file cr2017113x7.pdf]

Figure S7

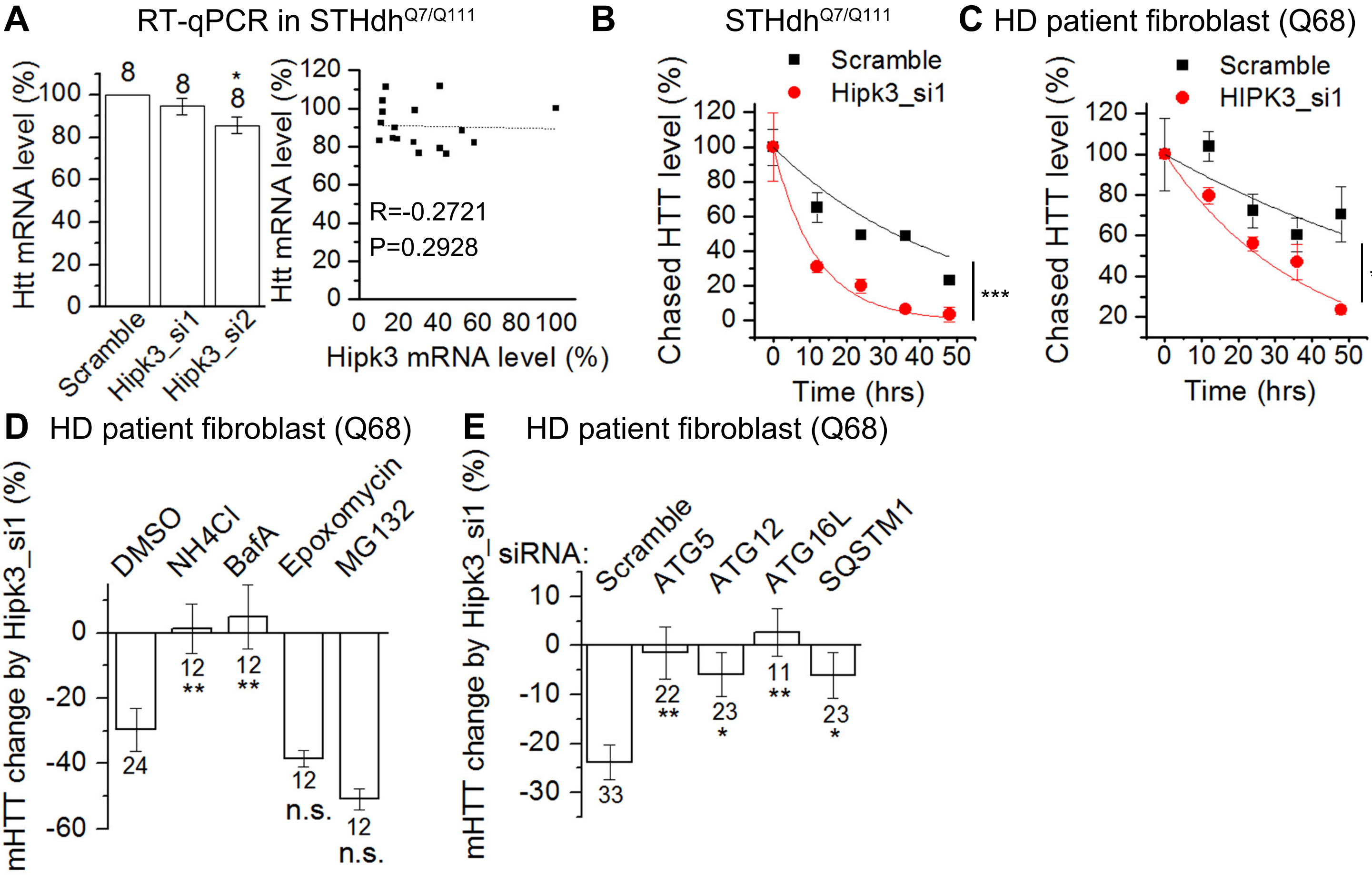

Figure S7 - Supplementary to Figure 6: Hipk3 regulates HTT levels via autophagy.

(A) *Left*: RT-qPCR in STHdh cells showing that Hipk3 knock-down causes no or mild lowering of the Htt mRNA levels. Statistical analysis by one-way ANOVA followed by Dunnett's post hoc tests. *Right*: scatter plots of Htt versus Hipk3 mRNA levels. The correlation coefficient R and P value are calculated by Spearman correlation analysis.

(B-C) Degradation curves measured by the non-radioactive pulse-chase assay of the STHdhQ7/Q111 cells (B) or immortalized HD patient fibroblasts Q68 (C) transfected with the indicated siRNAs. Hipk3 knock-down (by Hipk3 siRNA1) increases HTT degradation in both STHdhQ7/Q111 cells and HD patient fibroblasts (Q68). Degradation curves were fitted by exponential decay. Statistical analysis by two-way ANOVA (n = 3).

(D-E) mHTT lowering detected in HD patient fibroblasts by HTRF using the antibody pair 2B7/MW1, after treatment of compound inhibitors of proteasome or autophagy (D) or knock-down of autophagy genes in the patient HD fibroblasts (E). We have ensured that the treatment at the concentrations and duration applied did not cause signficant cell death, as tested by microscopic observation and total protein concentration measrements. Statistical analysis by one-way ANOVA followed by Dunnett's post hoc tests.

For all plots with error bars, data represent mean and SEM., and the numbers indicate biological replicates. "***": P<0.001; "**": P<0.01; "*": P<0.05;
