# Supplementary material for: Suppression of MAPK11 or HIPK3 reduces mutant Huntingtin levels in Huntington's disease models
Source: Cell Res. 2017 Oct 13;27(12):1441–65. doi: 10.1038/cr.2017.113 (PMC5717400; doi:10.1038/cr.2017.113)
Supplement: Supplementary information, Figure S8 — Rationale for using homozygous Q140 mice for phenotypic validation of Mapk11 [file cr2017113x8.pdf]

Figure S8

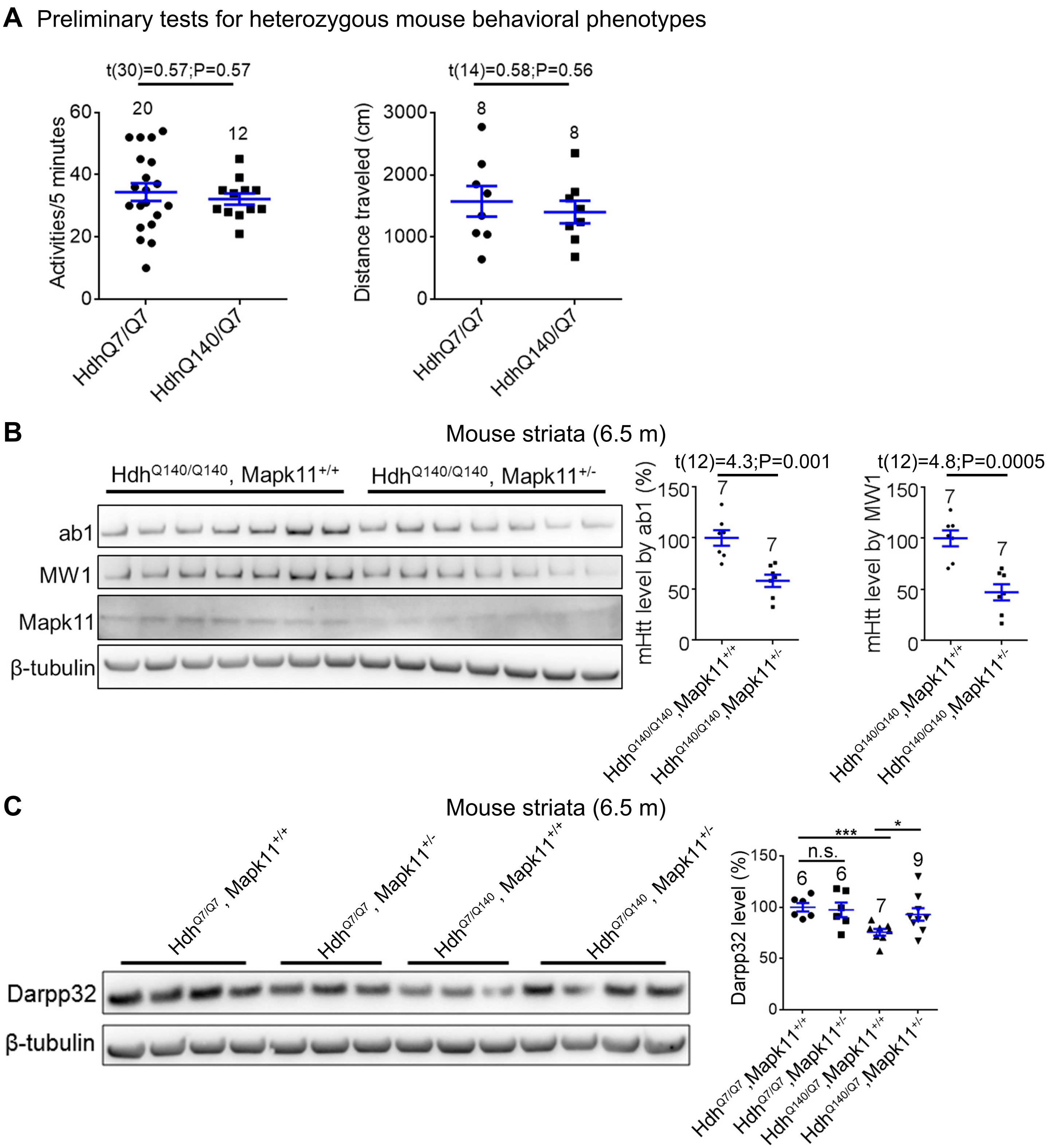
A
Preliminary tests for heterozygous mouse behavioral phenotypes
t(30)=0.57;P=0.57
Activities/5 minutes
20
12
HdhQ7/Q7
HdhQ140/Q7
t(14)=0.58;P=0.56
Distance traveled (cm)
8
8
HdhQ7/Q7
HdhQ140/Q7
B
Mouse striata (6.5 m)
HdhQ140/Q140, Mapk11+/+
HdhQ140/Q140, Mapk11+/-
ab1
MW1
Mapk11
β-tubulin
t(12)=4.3;P=0.001
mHtt level by ab1 (%)
t(12)=4.8;P=0.0005
mHtt level by MW1
HdhQ140/Q140,Mapk11+/+
HdhQ140/Q140,Mapk11+/-
C
Mouse striata (6.5 m)
HdhQ7/Q7, Mapk11+/+
HdhQ7/Q7, Mapk11+/-
HdhQ7/Q140, Mapk11+/+
HdhQ7/Q140, Mapk11+/-
Darpp32
β-tubulin
***
*
n.s.
Darpp32 level (%)
HdhQ7/Q7,Mapk11+/+
HdhQ7/Q7,Mapk11+/-
HdhQ140/Q7,Mapk11+/+
HdhQ140/Q7,Mapk11+/-

Figure S8-supplementary to Figure 8: rationale for using homozygous Q140 mice for phenotypic validation of Mapk11

(A) Preliminary behavioral testing results showing that the HdhQ7/Q140 heterozygous HD mice have very weak phenotypes to be assayed with in the activity and open-field tests for the potential rescue effect of lowering Mapk11. The numbers on top indicate the numbers of individual mice (~7.5m) tested. Unpaired two-tailed t tests were performed for statistical analysis, and the t and P values are shown on the top. Blue lines indicated mean and SEM.

(B) Western-blots and quantification (normalized to β-tubulin) showing that Mapk11$^{+/-}$ significantly reduced mHtt levels in the HdhQ140/Q140 homozygous mouse striata. Statistical analysis was performed by unpaired two-tailed t tests. Blue lines indicate mean and SEM.

(C) *Left* panels: representative western-blots of mouse striata samples of the indicated genotypes. *Right* panel: quantification of the Darpp32 signals normalized to the WT mice. Darpp32 was significantly lower in HD mice (3rd versus 1st bar), and this was rescue by heterozygous knockout of Mapk11 (4th versus 3rd bar). This rescue was HD-relevant because heterozygous knockout of Mapk11 did not influence Darpp32 levels in the wild-type background (2nd versus 1st bar). Data were plotted as mean and SEM; "***": $P < 0.001$, "*": $P < 0.05$, by unpaired two-tailed t tests for each pair of the compared groups. ANOVA was not used here because each comparison tests a different statistical hypothesis.
