# Supplementary material for: Suppression of MAPK11 or HIPK3 reduces mutant Huntingtin levels in Huntington's disease models
Source: Cell Res. 2017 Oct 13;27(12):1441–65. doi: 10.1038/cr.2017.113 (PMC5717400; doi:10.1038/cr.2017.113)
Supplement: Supplementary information, Table S1 — Component Score Coefficient Matrix [file cr2017113x9.pdf]

Table S1

A Component Score Coefficient Matrix

Component	λ	Weight (%)	Accumulative Weight
1	7.314	20.317	20.317
2	5.717	15.882	36.199
3	3.481	9.67	45.869
4	3.276	9.099	54.968
5	2.761	7.67	62.639
6	2.194	6.096	68.734
7	1.818	5.049	73.783
8	1.408	3.911	77.695
9	1.349	3.748	81.443
10	1.09	3.027	84.47
11	1.029	2.859	87.328
12	0.842	2.339	89.668
13	0.661	1.836	91.504
14	0.526	1.462	92.966
15	0.461	1.281	94.247
16	0.322	0.894	95.141
17	0.311	0.863	96.005
18	0.265	0.737	96.741
19	0.256	0.71	97.451
20	0.228	0.633	98.084
21	0.177	0.492	98.576
22	0.155	0.431	99.007
23	0.091	0.253	99.26
24	0.069	0.191	99.451
25	0.054	0.15	99.601
26	0.047	0.132	99.733
27	0.036	0.1	99.833
28	0.027	0.075	99.908
29	0.015	0.04	99.948
30	0.009	0.026	99.975
31	0.005	0.013	99.987
32	0.003	0.009	99.997
33	0.001	0.003	99.999
34	0.000	0.001	100
35	1.46E-05	4.06E-05	100
36	4.74E-16	1.32E-15	100

B

Principle Component 1 Coefficients

**Coupling parameter names**	**coefficient**
Couplings_RF->LH_Mean	-0.03
Couplings_RF->LH_CStat_Mean	-0.056
Couplings_RF->LH_CStat_R	0.094
Couplings_LF->RH_Mean	-0.033
Couplings_LF->RH_CStat_Mean	-0.096
Couplings_LF->RH_CStat_R	0.064
Couplings_LH->RF_Mean	-0.018
Couplings_LH->RF_CStat_Mean	0.044
Couplings_LH->RF_CStat_R	0.028
Couplings_RH->LF_Mean	0.032
Couplings_RH->LF_CStat_Mean	-0.003
Couplings_RH->LF_CStat_R	0.075
Couplings_LH->RH_Mean	0.063
Couplings_LH->RH_CStat_Mean	0.07
Couplings_LH->RH_CStat_R	0.058
Couplings_LF->RF_Mean	-0.069
Couplings_LF->RF_CStat_Mean	-0.062
Couplings_LF->RF_CStat_R	0.056
Couplings_RH->LH_Mean	-0.047
Couplings_RH->LH_CStat_Mean	-0.047
Couplings_RH->LH_CStat_R	0.07
Couplings_RF->LH_Mean	0.067
Couplings_RF->LH_CStat_Mean	0.069
Couplings_RF->LH_CStat_R	0.055
Couplings_RF->RH_Mean	0.063
Couplings_RF->RH_CStat_Mean	0.099
Couplings_RF->RH_CStat_R	0.063
Couplings_LF->LH_Mean	-0.064
Couplings_LF->LH_CStat_Mean	-0.065
Couplings_LF->LH_CStat_R	0.046
Couplings_RH->RF_Mean	-0.104
Couplings_RH->RF_CStat_Mean	-0.065
Couplings_RH->RF_CStat_R	0.063
Couplings_LH->LF_Mean	0.028
Couplings_LH->LF_CStat_Mean	0.065
Couplings_LH->LF_CStat_R	0.044

Table S1 – supplementary to Figure 8: Calculation of the principle component 1 for gait analysis
(A) The component score coefficient matrix calculated by SPSS based on the data obtained from all the

mice tested by the CatWalk gait analysis system (Figure 8F). The top component (principle component 1, λ=7.314) is used for the measurement of the HD-relevant gait phenotypes. The other major principle components (2, 4, 5 and 6) have no significant difference between HD and WT mice, whereas the principle component 3 has much smaller difference between HD and WT mice compared to the principle component 1, and is not rescued by Mapk11 knockouts.
(B) Coefficients of the principle component 1(PC1) used for the calculation for Figure 8F. PC1 is calculated as the weighted sum of the raw value of each coupling parameter.
