# Supplementary material for: Suppression of MAPK11 or HIPK3 reduces mutant Huntingtin levels in Huntington's disease models
Source: Cell Res. 2017 Oct 13;27(12):1441–65. doi: 10.1038/cr.2017.113 (PMC5717400; doi:10.1038/cr.2017.113)
Supplement: Supplementary information, Table S2 — Supplementary to Figure 8: gait analysis using single parameters [file cr2017113x10.pdf]

Table S2

Parameter	$Hdh^{Q7/Q7}$ Mapk11$^{+/+}$	$Hdh^{Q7/Q7}$ Mapk11$^{+/-}$	$Hdh^{Q7/Q7}$ Mapk11$^{-/-}$	$Hdh^{Q140/Q140}$ Mapk11$^{+/+}$	$Hdh^{Q140/Q140}$ Mapk11$^{+/-}$	$Hdh^{Q140/Q140}$ Mapk11$^{-/-}$
RF_StrideLength_(cm)_Mean	5.94 ± 0.31	5.91 ± 0.36	4.87 ± 0.21	5.24 ± 0.22	4.93 ± 0.24	5.48 ± 0.2
RH_MaxContactArea_(cm²)_Mean	0.42 ± 0.04	0.39 ± 0.04	0.47 ± 0.08	0.34 ± 0.03	0.47 ± 0.02	0.35 ± 0.04
RH_PrintArea_(cm²)_Mean	0.52 ± 0.04	0.5 ± 0.05	0.56 ± 0.09	0.43 ± 0.03	0.55 ± 0.03	0.43 ± 0.04
RH_StepCycle_(s)_Mean	0.72 ± 0.15	0.64 ± 0.17	0.76 ± 0.12	0.45 ± 0.03	0.57 ± 0.06	0.47 ± 0.05
RH_InitialDualStance_(s)_Mean	0.11 ± 0.02	0.06 ± 0.02	0.16 ± 0.04	0.06 ± 0.01	0.13 ± 0.03	0.1 ± 0.04
LH_MaxContactMeanIntensity_Mean	89.59 ± 1.9	79.1 ± 2.13	84.08 ± 1.99	79.86 ± 1.63	81 ± 1.6	84.55 ± 0.75
LH_PrintArea_(cm²)_Mean	0.54 ± 0.04	0.59 ± 0.06	0.74 ± 0.07	0.46 ± 0.04	0.63 ± 0.04	0.5 ± 0.05
PhaseDispersions_RF->RH_CStat_R	0.9 ± 0.04	0.96 ± 0.02	0.87 ± 0.11	0.8 ± 0.06	0.87 ± 0.05	0.89 ± 0.06
Couplings_RF->LH_CStat_Mean	31.92 ± 9.43	27.49 ± 18.29	54.49 ± 17.59	14.29 ± 4.91	23.74 ± 10.55	22.49 ± 12.16
Couplings_LF->LH_Mean	58.85 ± 4.62	51.42 ± 1.5	55.48 ± 2.28	51.67 ± 1.87	52.19 ± 1.45	53.63 ± 1.61
Couplings_RH->RF_CStat_Mean	49.74 ± 3.84	52.35 ± 3.8	43.95 ± 2.11	41.26 ± 2.61	46.35 ± 1.13	49.22 ± 3.05
Run_Maximum_Variation_(%)	66.16 ± 6.94	70.4 ± 15.79	64.43 ± 11.43	80.31 ± 5.46	71.41 ± 8.49	49 ± 8.32
RF_MaxContactAt_(%)_Mean	37.67 ± 1.98	47.17 ± 4.95	45.46 ± 2.75	46.28 ± 1.5	47.38 ± 3.33	42.73 ± 4.14
RF_MaxIntensityAt_(%)_Mean	52.72 ± 3.55	49.33 ± 3.53	50.52 ± 2.91	59.65 ± 2.68	54.39 ± 2.26	51.9 ± 3.15
RH_MaxIntensityAt_(%)_Mean	42.66 ± 3.59	46.61 ± 2.24	49.11 ± 5.14	52.98 ± 2.68	47.57 ± 2.53	50.59 ± 3.95
LF_MaxIntensityAt_(%)_Mean	52.2 ± 3.01	58.65 ± 1.44	53.08 ± 2.72	59.29 ± 2.46	51.41 ± 1.51	45.98 ± 2.75
LF_SwingSpeed_(cm/s)_Mean	32.03 ± 2.69	35.86 ± 3.69	33.91 ± 3.96	36.62 ± 2.12	31.58 ± 3.13	35.83 ± 1.57
LH_MaxContactAt_(%)_Mean	27.04 ± 2.09	26.34 ± 2.51	27.54 ± 2.97	33.03 ± 2.5	27.23 ± 2.56	23.19 ± 1.58
LH_MaxIntensityAt_(%)_Mean	41.51 ± 2.79	47.9 ± 4.5	47.47 ± 5.03	49.32 ± 3.12	43.63 ± 2.73	48.38 ± 2.24
StepSequence_NumberOfPatterns	1.91 ± 0.16	2.2 ± 0.2	2.86 ± 0.26	2.5 ± 0.16	2.91 ± 0.34	2.56 ± 0.22
OtherStatistics_NumberOfSteps	13.69 ± 0.56	14.6 ± 1.44	16.29 ± 0.75	15.75 ± 0.66	16.09 ± 1.19	14.5 ± 0.57
OtherStatistics_Cadence	6.64 ± 0.46	7.32 ± 1.01	5.36 ± 0.86	7.59 ± 0.42	6.47 ± 0.55	8.28 ± 0.83
PhaseDispersions_LF->RH_Mean	7.72 ± 2.07	6.33 ± 3.83	10.8 ± 2.83	17.17 ± 5.32	6.57 ± 2.67	10.35 ± 5.32
Couplings_LH->RF_CStat_Mean	55.41 ± 9.93	26.87 ± 18.17	55.21 ± 16.77	81.2 ± 6.99	69.09 ± 11.23	64.94 ± 14.11
Couplings_RH->LF_Mean	67.73 ± 7.82	92.67 ± 4.49	92.63 ± 1.58	84.9 ± 4.81	94.09 ± 3.01	91.72 ± 3.41
Couplings_LH->RH_CStat_Mean	48.3 ± 2.7	53.61 ± 1.59	54.38 ± 2.91	55.59 ± 3.75	50.94 ± 1.85	51.45 ± 1.67

Table S2-Supplementary to Figure 8: gait analysis using single parameters

Among the 214 parameters measured by for gait experiments, we selected all the 26 parameters that showed at least 10% difference between HD and WT mice with marginal (0.05<P<0.2) or significant (P<0.05, highlighted in yellow) P values by unpaired two-tailed t test. We then tested whether a certain parameter is rescued (green) or exacerbated (orange) by comparing $Mapk11^{+/-}$ or $Mapk11^{-/-}$ with $Mapk11^{+/+}$ in the same Htt background. The judgment was purely based on the direction of the difference between the averages of the compared groups. Data presented as mean ± SEM in every block.
