# Supplementary material for: Suppression of MAPK11 or HIPK3 reduces mutant Huntingtin levels in Huntington's disease models
Source: Cell Res. 2017 Oct 13;27(12):1441–65. doi: 10.1038/cr.2017.113 (PMC5717400; doi:10.1038/cr.2017.113)
Supplement: Supplementary information — Data S1 [file cr2017113x11.pdf]

Data S1

siRNA order information or target sequences:
Scrambled siRNA: non-targeting siRNA (Thermoscientific Dharmacon)
Mapk11_si1: ON-TARGETplus Mouse Mapk11 SMARTpool (Thermoscientific Dharmacon, L-050928-00-0005)
Mapk11_si2: Silencer® Select siRNA (Thermofisher Ambion, #s72151, targeting 5'- CGCCAGAGAUCAUGCUAAATT-3')
Map2k6_si: ON-TARGETplus Mouse Map2k6 SMARTpool (Thermoscientific Dharmacon, L-043421-00-0005)
MAPK11_si_1: 5'- CGCAUGUAUGCAUGCACAATT-3'
MAPK11_si_2: 5'- GAACACGCCCGGACAUAUATT-3'
MAP2K6_si1: ON-TARGETplus Human MAPK11 SMARTpool (Thermoscientific Dharmacon)
MAP2K6_si2: FlexiTube siRNA pool (Qiagen, #GS5608)
ATG5_si: 5'- GCCUGUAUGUACUGCUUUA-3';
SQSTM1_si: 5'-GAUCUGCGAUGGCUGCAAU-3';
ATG12_si: 5'-GAACACCAAGUUUCACUGU-3';
ATG16L-si: 5'-UGUGGAUGAUUAUCGAUUA-3';
DAXX-si: 5'- GGAGUUGGAUCUCUCAGAA-3';
HTT siRNA (HTT3): 5'-CAGGUUUAUGAACUGACGUUA-3'
Htt siRNA (B01): 5'-CUCAUUGUGAAUCACAUUCAA-3'
Htt siRNA (C01): 5'-CUGGUUGGUAUUCUUCUAGAA-3'
Htt siRNA (Hdh5): 5'-ACCGUCUCAAUCAUUGUCUAA-3'

qPCR primer sequences (5'~3', all primers have been validated by standard curves for efficiency (95%~105%) and linearity (R^2>0.999):

Hprt-F	GTCAACGGGGGACATAAAAG
Hprt-R	CAACAATCAAGACATTCTTTCCA
Htt-F	CTGCACGGCATCCTCTATGT
Htt-R	TGTTCACGCAGTGGGCTATT
Htt(#38)-F (for Htt mRNA stability)	CCTTCCCGTGAGTGAGTGA
Htt(#38)-R (for Htt mRNA stability)	GCTGATAAAACCTGTCCCATGA
Map2k6-F	CGGCCCACATATCCAGAG
Map2k6-R	ATGGCTTTTTAGTCCCCAAGT
Mapk11-F	CCTGAGGTTCTGGCAAAGAT
Mapk11-R	CACTGCTGAGGTCCTTCTGG
HPRT-F	CCTGGCGTCGTGATTAGTG
HPRT-R	TGATTAATAAACACCCTTTCCA
HTT-F	CCTTTTTGAAGCAGCCCGTG
HTT-R	TGCAGCATCCCCAAACAGAT
DAXX_F	GAAGACGATGAGGAGAGTGATGAG
DAXX_R	GGCTCTTGTCTCCATCTTTACCTG
